# Exploring a Nitric Oxide-Releasing Celecoxib Derivative as a Potential Modulator of Bone Healing: Insights from Ex Vivo and In Vivo Imaging Experiments

**DOI:** 10.3390/ijms26062582

**Published:** 2025-03-13

**Authors:** Christin Neuber, Luisa Niedenzu, Sabine Schulze, Markus Laube, Frank Hofheinz, Stefan Rammelt, Jens Pietzsch

**Affiliations:** 1 Department Radiopharmaceutical and Chemical Biology, Institute of Radiopharmaceutical Cancer Research, Helmholtz-Zentrum Dresden-Rossendorf, Bautzner Landstrasse 400, 01328 Dresden, Germany; m.laube@hzdr.de (M.L.); j.pietzsch@hzdr.de (J.P.); 2 University Center for Orthopaedics, Trauma and Plastic Surgery, University Hospital Carl Gustav Carus at Technische Universität Dresden, Fetscherstrasse 74, 01307 Dresden, Germanysabine.schulze@tu-dresden.de (S.S.); stefan.rammelt@ukdd.de (S.R.); 3 Department Positron Emission Tomography, Institute of Radiopharmaceutical Cancer Research, Helmholtz-Zentrum Dresden-Rossendorf, Bautzner Landstrasse 400, 01328 Dresden, Germany; f.hofheinz@hzdr.de; 4 Faculty of Chemistry and Food Chemistry, School of Science, Technische Universität Dresden, Bergstraße 66, 01069 Dresden, Germany

**Keywords:** bone healing, inflammation, cyclooxygenase-2, revascularization, molecular imaging, small animal imaging, positron emission tomography, dual-radiotracer approach, rat critical bone defect

## Abstract

The inducible enzyme cyclooxygenase-2 (COX-2) and the subsequent synthesis of eicosanoids initiated by this enzyme are important molecular players in bone healing. In this pilot study, the suitability of a novel selective COX-2 inhibitor bearing a nitric oxide (NO)-releasing moiety was investigated as a modulator of healing a critical-size bone defect in rats. A 5 mm femoral defect was randomly filled with no material (negative control, NC), a mixture of collagen and autologous bone fragments (positive control, PC), or polycaprolactone-co-lactide (PCL)-scaffolds coated with two types of artificial extracellular matrix (aECM; collagen/chondroitin sulfate (Col/CS) or collagen/polysulfated hyaluronic acid (Col/sHA3)). Bone healing was monitored by a dual-tracer ([^18^F]FDG/[^18^F]fluoride) approach using PET/CT imaging in vivo. In addition, ex vivo µCT imaging as well as histological and immunohistochemical studies were performed 16 weeks post-surgery. A significant higher uptake of [^18^F]FDG, a surrogate marker for inflammatory infiltrate, but not of [^18^F]fluoride, representing bone mineralization, was observed in the implanted PCL-scaffolds coated with either Col/CS or Col/sHA3. Molecular targeting of COX-2 with NO-coxib had no significant effect on tracer uptake in any of the groups. Histological and immunohistochemical staining showed no evidence of a positive or negative influence of NO-coxib treatment on bone healing.

## 1. Introduction

Although autologous bone grafting is still the clinical gold standard for treatment of large bone defects, this approach is limited by the need of an additional surgery, availability of donor material, and donor site morbidity [1,2]. Therefore, various tissue engineering strategies aim to develop biomaterials providing an environment mimicking the body’s natural bone healing process [3], e.g., by promoting osteogenesis and angiogenesis, by facilitating integration with host tissues and load transfer under weight-bearing conditions [2]. Finally, such materials should be bioresorbable [2]. All of these requirements are largely fulfilled by polycaprolactone-co-lactide (PCL)-scaffolds coated with collagen type I (Col), the most abundant organic matrix component of bone, and the glycosaminoglycans (GAGs) chondroitin sulfate (CS) or hyaluronan (HA), which are important multifunctional components of the extracellular matrix (ECM) in bone, as demonstrated by previous studies [4,5,6]. The latter stimulate bone healing by recruiting mesenchymal stromal cells and supporting their differentiation [5,7]. As the underlying mechanism, binding of growth factors and cytokines and, thereby, modulation of their availability has been discussed [5,8]. The positive effect of such coated implants has already been demonstrated both in vitro [9,10,11,12] and in vivo [5,6,13,14,15], suggesting that the investigated scaffolds could act not just as temporary matrix for cell migration, proliferation, and differentiation in bone tissue engineering, but also have a great potential as bone substitutes [14].

During treatment of bone defects, especially in the early phase of bone healing, sufficient pain management is indispensable in view of patient welfare. In this regard, nonsteroidal anti-inflammatory drugs (NSAIDs) like aspirin, ibuprofen, or naproxen sodium are highly effective against fever, pain, and inflammation by blocking constitutively expressed cyclooxygenase (COX)-1 and inducible COX-2. In comparison to NSAIDs, COX-2 selective inhibitors (coxibs) are characterized by a reduced risk of adverse side effects with regard to stomach, intestine, and kidney function and, thereby, are to be preferred over non-selective COX inhibitors at least in patients without pre-existing vascular risk factors. However, therapeutic modulation of COX-2 function is discussed controversially with regard to bone healing, especially during its early phase [16], and seems to be challenging due to the narrow therapeutic window. Nevertheless, COX-2 represents a key factor in inflammatory processes accompanying bone healing [16,17,18,19] and, therefore, this enzyme itself is an excellent molecular target, as it is strongly expressed primarily in the immediate vicinity of the local defect. This provides the opportunity to use coxibs as targeting units to pursue further therapeutic approaches.

Alongside inflammation, angiogenesis is crucial for proper bone healing [20,21]. Besides other factors, the small, hydrophobic, and highly reactive free radical NO• (nitric oxide; NO), produced by upregulated inducible (iNOS) and endothelial NO synthases (eNOS) in osteoblasts and periosteal cells, facilitates neovascularization during bone healing by vasodilatation as well as by delivery of inflammatory cells and cytokines to the fracture site [22,23,24]. Moreover, NO mediates bone remodeling and stimulates the production of collagen precursors [20,23,25,26]. Therefore, local delivery of NO as a modulator of both bone healing and vascular reactivity represents an area of growing therapeutic potential [20]. The effects of both coxibs and NO alone on bone healing have been extensively reviewed elsewhere [16,21].

We hypothesized that the targeting of COX-2 for the delivery of NO to the fracture site positively influences (biomaterial-assisted) bone healing. To prove our hypothesis, we used the potent NO-releasing COX-2 inhibitor [4-Chloro-1-(4-sulfamoylphenyl)-5-(p-tolyl)-1*H*-pyrazol-3-yl]methyl nitrate [27], which was previously developed by our group [28] and is referred to as NO-coxib herein, for adjuvant treatment of a critical-size bone defect in rats. The structure of NO-coxib is based on the clinically approved COX-2 inhibitor celecoxib and contains a nitroester moiety at the central pyrazole heterocycle as NO-releasing functionality. To investigate the efficacy of this approach, various bone healing scenarios, reflecting different clinical situations, were used as models. While the empty defect serves as negative control (NC), a mixture of porcine gelatine consisting of collagen and sodium lauryl sulfate together with autologous bone fragments represents a positive control (PC). Both were compared to PCL-scaffolds coated with a mixture of collagen type I and CS (Col/CS) or high-sulfated HA (Col/sHA3), which are important multifunctional components of the ECM in bone.

Bone healing was investigated intraindividually over 16 weeks by dual-radiotracer PET/CT imaging using the clinically established radiotracers 2-[^18^F]fluoro-2-deoxy-D-glucose ([^18^F]FDG) and [^18^F]fluoride. Positron emission tomography/computed tomography (PET/CT) imaging is particularly suitable for such longitudinal studies as it allows for non-invasive imaging of the target of interest, which can be, depending on the type of used radiotracer, expression of a certain receptor, activity of transporters or enzymes, or a metabolic process [29,30]. Both the animal model for biomaterial-assisted bone defect healing and feasibility of the dual-radiotracer PET/CT imaging for functional characterization of biomaterial-assisted bone healing have already been validated [6]. Finally, explanted femora were analyzed by ex vivo CT, histology, and immunohistochemistry to obtain insights into bone healing, remodeling, vascularization, and the presence of osteoblasts, osteoclasts, as well as inflammatory cells.

To the best of our knowledge, this is the first preclinical study investigating biomaterial-assisted bone healing in combination with a novel NO-coxib by non-invasive PET/CT imaging.

## 2. Results

### 2.1. First In Vivo Application of NO-Coxib

The first in vivo application of the NO-coxib developed by our group [27,28] was well-tolerated by the animals. No adverse side effects were observed in male Wistar rats when applying 10 doses within 14 days with each 5 mg NO-coxib/kg body weight by s.c. injection in a bio-compatible vehicle solution (50% PEG300, 40% NaCl, 10% DMSO containing NO-coxib; 1 mL/kg body weight).

### 2.2. Imaging of Inflammatory Activity by [^18^F]FDG PET/CT

Cellular activity in the bone defect area was investigated by PET/CT imaging after i.v. injection of [^18^F]FDG. [^18^F]FDG accumulation in tissues is mainly dependent on cellular glucose uptake by glucose transporters (GLUT) 1 and 3 [31]. Thereby, it is a surrogate marker for cellular glucose consumption and, in line with this, cellular activity. Besides tumor cells (“Warburg effect” [32,33]), inflammatory cells also have a high need for glucose [34], whereas no significant uptake of [^18^F]FDG is observed in healthy bone with the exception of epiphyses [6].

**Figure 1:**
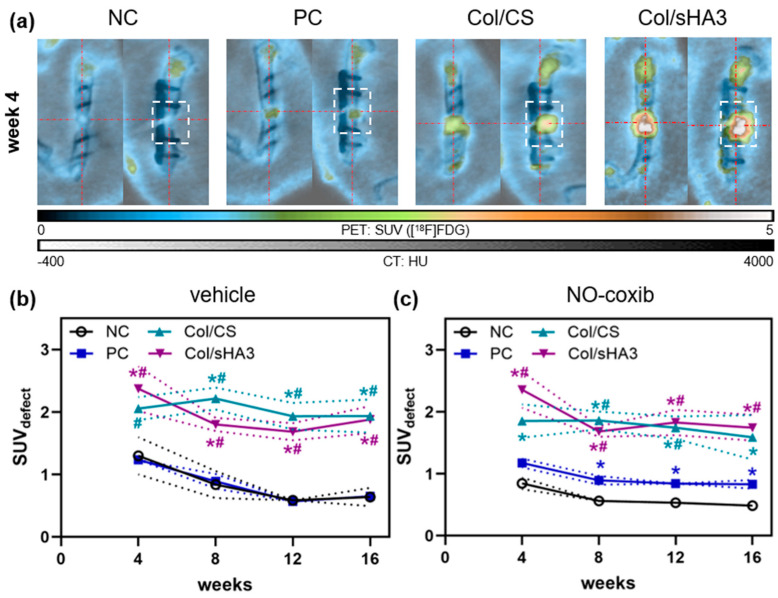
[^18^F]FDG accumulation in the femur defect after insertion of no material (NC), a mixture of collagen and autologous bone fragments (PC), or PCL-scaffolds coated with a mixture of either collagen and chondroitin sulfate (Col/CS) or collagen and polysulfated hyaluronic acid (Col/sHA3). (**a**) Representative PET/CT images at 4 weeks post-surgery. (**b**,**c**) [^18^F]FDG accumulation in the bone defect (dashed box) was determined (SUV_defect_) at 4, 8, 12, and 16 weeks post-surgery without ((**b**), vehicle solution only) or with adjuvant administration of NO-coxib (**c**). All values are depicted as mean (symbol) ± SEM (dotted line). Significant differences to the NC and PC are indicated by * and #, respectively (*p* ≤ 0.05).

Four weeks after establishment of the 5 mm femur defect and implantation of the biomaterials, PET/CT imaging was conducted the first time. At that time, [^18^F]FDG predominantly accumulated within the bone defect, especially when an aECM-coated PCL-scaffold was implanted (Figure 1a, dashed box). In comparison to the NC and PC, a substantial higher [^18^F]FDG uptake was observed in the implanted PCL-scaffolds (Col/CS and Col/sHA3) (Figure 1a). Considerable [^18^F]FDG uptake in aECM-coated PCL-scaffolds suggests an increased glucose consumption due to increased cellular activity within these scaffolds, which indicates a prominent infiltration by inflammatory cells.

For quantification of tracer uptake, standard uptake value (SUV) was determined in a volume of interest (VOI) comprising the bone defect and adjacent bone tissue, which was defined as SUV_defect_ (Figure 1a, dashed box). Determination of SUV_defect_ between the two inner screws is superior to analyzing the whole area between the two outer screws due to enhanced physiological [^18^F]FDG uptake by epiphyses resulting in overestimation of inflammation-induced tracer uptake [6]. SUV values were determined at 4, 8, 12, and 16 weeks post-surgery to generate time activity curves (TAC, Figure 1b) over the whole observation period. Thereby, it became evident that, beginning with latest week 8 post-surgery, there is a significantly higher [^18^F]FDG uptake by bone defects filled with aECM-coated PCL-scaffolds in comparison to NC and PC (Figure 1b). In NC, PC, and Col/sHA3 groups, the highest [^18^F]FDG uptake was found at week 4 with a slight decrease thereafter followed by a stable phase up to at least week 16 (end of observation period) (Figure 1b), suggesting inflammatory processes to be most active in the bone defect up to 4 weeks post-surgery. Only in rats implanted with Col/CS the maximum [^18^F]FDG uptake was observed slightly later, at week 8, followed by a fairly stable tracer uptake up to week 16 (Figure 1b). Analyzing area under the (time activity) curve (AUC_SUV_) revealed significantly higher total [^18^F]FDG uptake in rats implanted with Col/CS (24.6 ± 2.6) or Col/sHA3 (22.5 ± 2.4) compared to NC (9.6 ± 1.8) or PC (9.6 ± 0.9) (Figure S1E). This confirms previous findings that the presence of GAGs, especially CS, stimulates recruitment, attachment, and proliferation of mesenchymal stem cells (MSCs) in vitro and cells from bone marrow and the surrounding defect area in vivo [10,14]. Moreover, CS induced osteogenic differentiation of human MSCs in vitro without other differentiation additives [10]. In the corresponding in vivo experiment, coating of PCL-scaffolds with Col/CS resulted in the highest matrix deposition and bone formation compared to non-coated PCL-scaffolds [14], suggesting that the investigated scaffolds do not just act as temporary matrix for cell migration, proliferation, and differentiation in bone tissue engineering, but also have an osteogenic potential as bone substitutes [14].

To test whether NO delivery to bone defect via COX-2 targeting influences bone defect inflammation and healing, rats were treated at 10 out of 14 days from day 2 to day 14 post-surgery with either NO-coxib (5 mg/kg per dose) or vehicle solution only by s.c. injection next to the surgery site. Compared to the vehicle group (Figure 1b), [^18^F]FDG uptake in NO-coxib treated rats showed mainly the same SUV levels and time courses (Figure 1c). Direct comparison within each group (Figure S1A–D) revealed only for PC group at 12 weeks post-surgery a significant difference between vehicle and NO-coxib-treated rats. By trend, a lower [^18^F]FDG uptake was observed in the NC group at week 4 and 8 post-surgery when rats where treated with NO-coxib (Figure S1A), suggesting a slightly decreased initial inflammation in the presence of NO-coxib in this group. Nevertheless, due to relatively high interindividual variations, no further significant differences between the vehicle and NO-coxib group could be observed. Although total [^18^F]FDG uptake over time (AUC_SUV_) did not significantly differ in any of the groups (Figure S1E), again there was a trend for a decreased [^18^F]FDG signal in rats implanted with no material (NC) when treated with NO-coxib (Figure S1E).

In order to reduce interindividual variations, various attempts have been made to normalize tracer signal in the bone defect to either “background” tracer uptake in the reference tissue muscle (target-to-background ratio; TBR) or to tracer input function in blood (standard uptake ratio; SUR). In addition, Patlak analysis has been performed to determine the metabolic uptake rate K_m_. However, none of the normalized values were superior to SUV with regard to data variation within the groups (Figure S2). With the exception of K_m_, all of them showed the same trends with regard to time course and differences between experimental groups. Both TBR and K_m_ confirmed a slightly, although not significantly, decreased [^18^F]FDG signal in the NC group when treated with NO-coxib in comparison to the vehicle group (AUC_NO-coxib_ vs. AUC_vehicle_: TBR: 22.1 ± 2.7 vs. 28.7 ± 5.7; K_m_: 0.06 ± 0.02 vs. 0.09 ± 0.02). This indicates that inflammatory activity in the non-filled femur defect seems to be slightly decreased by NO-coxib administration.

One reason for missing measurable effects of NO-coxib in rats of Col/CS and Col/sHA3 group could be that aECM-coated PCL-scaffolds already attract a variety of cells. Especially for Col/CS-coated scaffolds, it has already been discussed that they seem to have such good bone-healing effects that seeding with mesenchymal stem cells (MSCs) does not have an additional benefit, although their presence could be demonstrated even up to 12 weeks after implantation [14].

### 2.3. Imaging of Bone Mineralization by [^18^F]Fluoride PET/CT

Bone mineralization within the femur defect was analyzed by PET/CT imaging after i.v. injection of [^18^F]fluoride. [^18^F]Fluoride is transported to the skeletal system in proportion to bone blood flow and osteoblastic activity and, finally, accumulates as a function of fluorine-to-hydroxyapatite exchange [35,36].

**Figure 2:**
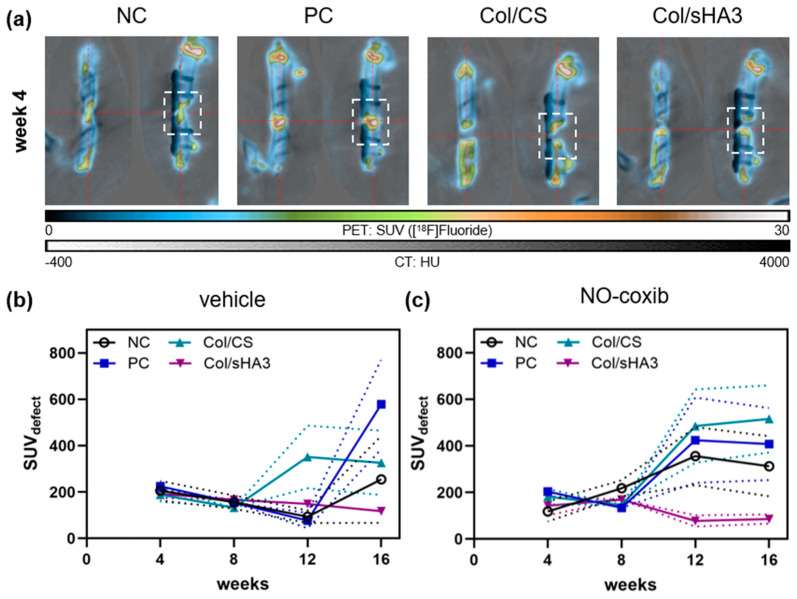
[^18^F]Fluoride accumulation in the femur defect after insertion of no material (NC), a mixture of collagen and autologous bone fragments (PC), or PCL-scaffolds coated with a mixture of either collagen and chondroitin sulfate (Col/CS) or collagen and polysulfated hyaluronic acid (Col/sHA3). (**a**) Representative PET/CT images at 4 weeks post-surgery. (**b**,**c**) [^18^F]Fluoride accumulation in the bone defect (dashed box) was determined (SUV_defect_) at 4, 8, 12, and 16 weeks post-surgery without ((**b**), vehicle solution only) or with adjuvant administration of NO-coxib (**c**). All values are depicted as mean (symbol) ± SEM (dotted line). No significant differences could be observed.

Due to the different targeting mechanisms, [^18^F]fluoride accumulates, contrary to [^18^F]FDG, at the bone defect margins in close proximity to the 5 mm femur defect, where highest new bone formation and mineralization is expected (Figure 2a). Only in the PC group, where a mixture of collagen and autologous bone fragments has been implanted, [^18^F]fluoride accumulation was found in the entire defect (Figure 2a). Obviously, autologous bone fragments allow for a more evenly distributed formation of new bone.

When analyzing TACs (Figure 2b), it became evident that, at 4 and 8 weeks post implantation, [^18^F]fluoride accumulation was comparable in all four groups and, with the exception of Col/CS, decreased from week 4 to 12. From 12 weeks forward, substantial differences between the different groups became apparent. While [^18^F]fluoride accumulation in NC and especially in PC was increased at week 16 post-surgery compared to week 12, suggesting an active bone formation at that time, [^18^F]fluoride signal in rats implanted with Col/CS was already increased at 12 weeks post-surgery, but afterwards stabilized (Figure 2b). By contrast, [^18^F]fluoride signal in rats implanted with Col/sHA3 continuously decreased from 4 weeks post-surgery, suggesting the lowest bone mineralization of all. Analyzing AUC_SUV_ from TACs (Figure S3E) revealed highest tracer uptake over the entire observation period in rats of Col/CS (2962 ± 1260) and PC group (2533 ± 889), whereas AUC_SUV_ for Col/sHA3 (1882 ± 176) was lower and comparable to NC (1917 ± 718). However, with the numbers available, no significant differences could be detected due to high interindividual variations.

Adjuvant treatment with NO-coxib (10 doses, 5 mg/kg per dose) did not significantly influence bone mineralization-related [^18^F]fluoride uptake in NC, PC, and Col/CS group (Figure S3A–D). However, at 12 weeks post-surgery, there was a trend towards a higher [^18^F]fluoride uptake in these groups when treated with NO-coxib (Figure S3A–C). This was also true for rats implanted with Col/CS at week 16 (Figure S3C), resulting in highest overall tracer uptake over time (AUC_SUV_ 3918 ± 1419, Figure S3E). This suggests that adjuvant treatment with NO-coxib might have positive effects on bone formation in non-filled defects (NC), but also in defects filled with autologous bone (PC) or a suitable biomaterial (Col/CS). Interestingly, these effects manifest mostly at later phases of bone healing (later than week 8 post-surgery), although they are supposed to be initiated in the early phase of bone healing because NO is a highly reactive free radical (NO•) with a correspondingly short half-life. By contrast, implantation of PCL-scaffolds coated with Col/sHA3 resulted in lowest overall bone formation (AUC 1882 ± 176), which was further significantly diminished by subcutaneous injection of NO-coxib next to the surgery site (AUC 1445 ± 323, *p* = 0.007) (Figure S3D,E).

According to [^18^F]FDG uptake, [^18^F]fluoride signal has been normalized to either “background” tracer uptake in the reference tissue muscle (TBR) or to tracer input function in blood (SUR, Patlak analysis). However, none of the normalized values were superior to SUV with regard to data variation within the groups.

### 2.4. Quantitative Analysis of Bone Defect Closure by High-Resolution CT and µCT Imaging

**Figure 3:**
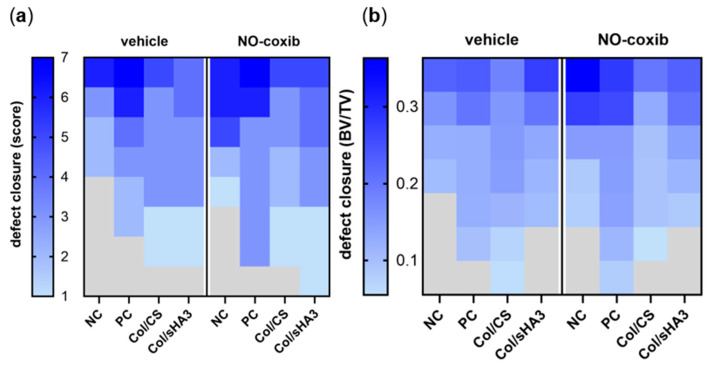
CT-based evaluation of defect bridging in a femur defect model 16 weeks post-surgery. (**a**) Directly after explantation, femora were imaged by high-resolution CT and defect bridging was quantified by defect closure score. (**b**) After fixation, femora were imaged by µCT and bone volume ratio (bone volume (BV)/total volume (TV)) was calculated with software.

In addition to functional PET imaging, newly formed bone volume as an endpoint marker was investigated at 16 weeks post-surgery, when rats passed all in vivo imaging experiments. Explanted femora were analyzed by both high-resolution CT (Figure S4A and Figure 3a) and µCT imaging (Figure S4B and Figure 3b) to finally assess bone formation within the femur defect. To avoid metal-based CT artifacts, plate and screws had to be removed before CT imaging even though this bears the risk of damage to the newly formed bone tissue. Especially for Col/CS, which tended towards bone bridging in close proximity to the plate, this often happened and, therefore, bone defect closure in the Col/CS group is likely to be underestimated by ex vivo CT imaging. Moreover, when bone bridging is still fragile and the femora are subject to bending, bone volume within the defect area determined by a computer-based method may be under- or overestimated. Therefore, we decided to determine bone healing by a simple scoring method (Figure S4C) in addition to image-based determination of ratio between bone volume (BV) and total volume (TV) (Figure S4D).

After 16 weeks, in all of the investigated groups, a healing of the femur defect could be observed. However, interindividual variation within the groups was quite high. While in two animals complete defect closure (score 7) could be observed, there were also 10 rats still without any defect bridging (score 1) (Figure 3a). Despite some significant differences and trends in [^18^F]FDG and [^18^F]fluoride uptake, respectively, there were no significant differences with respect to defect bridging between the groups, probably due to data variability within the groups regardless of the method used (Figure 3a,b). Even in the PC group, reflecting the current clinical gold standard, defect bridging varied from score 2 to 7. Implantation of PCL-scaffolds coated with Col/CS did not result in higher bone defect closure, as expected from the highest bone mineralization rates suggested by [^18^F]fluoride imaging. On the other hand, implantation of Col/sHA3-coated PCL-scaffolds, which was characterized by lowest [^18^F]fluoride uptake overall, did not result in a significantly reduced bone defect bridging compared to the other groups.

Treatment with NO-coxib did not show any measurable effects on defect bridging analyzed by high-resolution CT or µCT (Figure 3). However, there are some trends towards higher defect bridging in NO-treated rats. In general, there was a good correlation between determination of defect bridging by manual scoring (high-resolution CT) and software-based calculation (µCT) (Figure 3).

### 2.5. Qualitative Characterization of Bone Defect Closure by Histology

CT measurement only detects mineralized/calcified bone tissue and, thereby, may not recognize or at least underestimate defect bridging with cartilage and pre-mature bone tissue. Therefore, explanted femora were also investigated by histology and immunohistochemistry basically according to previously published methods [37].

In hematoxylin and eosin staining, mature bone, appearing as a compact structure in dark red, can be discriminated from connective tissue, displayed as a structured network of cells and collagen fibers in light pink, or cartilage, in gray-pink (Figure S5A). This allows for the newly formed tissue in the defect area to be examined for the amount of calcified bone and cartilage as well as the distribution of cells and blood vessels. Defect bridging in the NC group, for example, varied from still completely separated bone ends to almost complete bone defect closure with only a narrow strip of connective tissue, which is in line with the results obtained by ex vivo CT. Since rats were obtained by the same supplier and even as one cohort, this emphasizes individual variability in bone defect healing capacity of the used rats.

**Figure 4:**
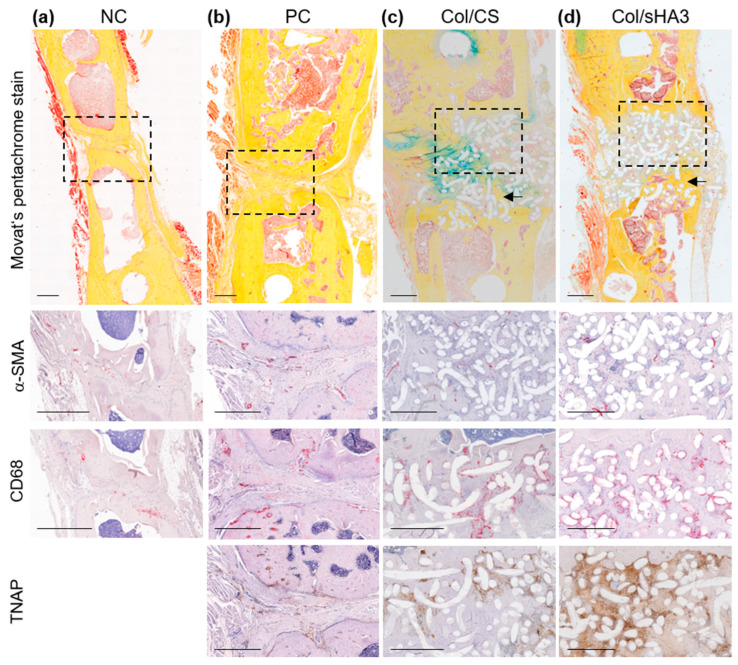
Representative images of Movat’s pentachrome stain and, in an enlarged section (dotted box), blood vascular endothelial cells (αSMA, alpha-smooth muscle actin), M1 macrophages (CD68), and osteoblasts (TNAP, tissue-nonspecific alkaline phosphatase) in femur defect 16 weeks after insertion of (**a**) no material (NC), (**b**) collagen together with autologous bone (PC), or a PCL-scaffold coated with (**c**) Col/CS or (**d**) Col/sHA3 (Col = collagen, CS = chondroitin sulfate, sHA3 = polysulfated hyaluronic acid). Mature bone within PCL-scaffolds is indicated by arrows. Bar = 1 cm.

Movat’s pentachrome staining allows for a more detailed analysis of bone healing progression as well as the type of bone formation (direct, or desmal, ossification vs. endochondral ossification) [37]. While mature bone can be identified by its compact structure in dark yellow, fibrous tissue and cartilage appear in light yellow and green, respectively (Figure S5B). Thereby, newly formed bone in NC and PC could be characterized as woven bone with bone marrow already (Figure 4a,b and Figure S5B). Implanted PCL-scaffolds, recognizable by the white spots in the histological sections [27], were for the most part still present in rats after 16 weeks (Figure 4c,d), which may delay bone healing in the used rat model. On the other hand, the degradation behavior of PCL-scaffolds is optimized for human application and is considered to be fully biodegradable in patients. Beforehand, aECM-coated PCL-scaffolds provide osteoconductive, osteoinductive, and osteogenic properties ([5] and references therein). While bone defect healing in NC and PC was characterized predominantly by either endochondrale (NC, Figure S6A) or direct ossification (PC, Figure S6B), bone defect bridging in rats implanted with Col/CS or Col/sHA3 occurred by both endochondral and direct ossification in parallel (Figure 4c and Figure S6C). Using Movat’s pentachrome staining, cells within the bone defect can be clearly identified by their red-brown nuclei staining in contrast to yellow collagen and, moreover, the active stage of bone formation can be verified by newly formed osteoid colored in red [37].

With Masson–Goldner trichrome staining, mature bone appears in dark blue to turquoise, connective tissue in light turquoise to green, and muscle as well as cytoplasm in red, providing again a good method to distinguish between areas of mineralized bone from premature bone or connective tissue (Figure S5C).

**Figure 5:**
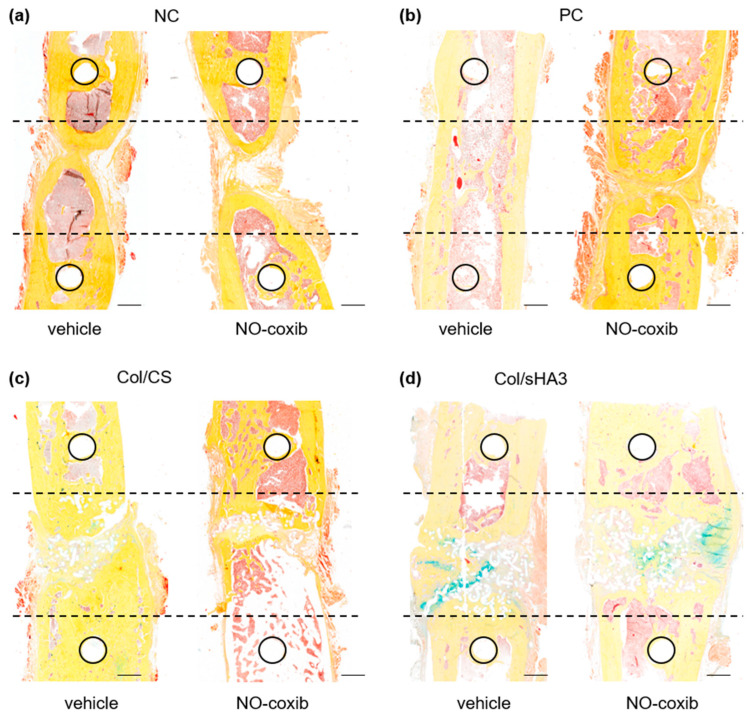
Representative images of Movat’s pentachrome stain comparing proportion of mature bone within the defect area 16 weeks after insertion of (**a**) no material (NC), (**b**) collagen together with autologous bone (PC), or a PCL-scaffold coated with (**c**) Col/CS or (**d**) Col/sHA3 (Col = collagen, CS = chondroitin sulfate, sHA3 = polysulfated hyaluronic acid) in rats treated with either vehicle or NO-coxib. Circles mark the previous position of the inner screws and dotted lines cover the original defect area. Bar = 1 cm.

By contrast to the trends found by in vivo PET/CT imaging using [^18^F]fluoride and ex vivo CT measurements, no clear differences between rats treated with NO-coxib or vehicle solution could be observed by histology (Figure 5). This is most likely caused by the limited number of histological specimens per group (n = 3–4) and interindividual variation, even in rats investigated within one experiment. Moreover, by contrast to in vivo PET and ex vivo CT imaging, histological characterization represents only one particular section of the bone defect and, therefore, may be less representative for assessment of complete bone defect bridging.

### 2.6. Qualitative Characterization of Bone Defect Closure by Immunohistochemistry

With immunohistochemistry, the presence of osteoblasts (Figure S5D; tissue-nonspecific alkaline phosphatase (TNAP)), osteoclasts (tartrate-resistant acid phosphatase, TRAP), macrophages type I (Figure S5E; CD68), macrophages type II (CD163), and blood vessels (Figure S5F; alpha-smooth muscle actin (α-SMA)) were analyzed.

At 16 weeks post-surgery, the highest TNAP activity could be found in the Col/CS and Col/sHA3 groups (Figure 4c,d). TNAP is an enzyme that plays a crucial role in bone formation and is produced by osteoblasts. TNAP increases the local concentration of inorganic phosphate by hydrolyzing molecules containing phosphate groups and, thereby, enhances precipitation of calcium phosphate, a key component of hydroxyapatite. In bone regeneration, TNAP expression is elevated, especially in the phase of soft callus formation and during the transition from soft to hard callus [38]. While osteoclasts and M2 macrophages were almost absent in the analyzed bone defect specimens, suggesting bone healing being not yet in the remodeling phase, M1 macrophages could be observed especially at the border line of bone and connective tissue (Figure 4a,b and Figure S5E).

As expected, advanced defect bridging could be observed in most of the analyzed rats from the PC group in contrast to the NC group. In PC, bone margins were in close proximity and there was only a narrow strip of connective tissue in between, pervaded by blood vessels, M1 macrophages, and osteoblasts (Figure 4b and Figure S5E,F). PCL-scaffolds coated with either Col/CS or Col/sHA3 were well-penetrated with connective tissue and, in most cases, only minor parts of the PCL-scaffold were surrounded by mature bone, as demonstrated by Masson–Goldner and Movats’ pentachrome staining (Figure 4c,d, arrows). In general, many osteoblasts could be found within the defect area (Figure 4c,d and Figure S5D), suggesting an active bone mineralization occurring, accompanied by the presence of M1 macrophages especially at the borderline of bone and connective tissue (Figure 4 and Figure S5E). In all groups, blood vessels could be observed within the defect area (Figure 4), mainly limited to areas of connective tissue.

However, due to the limited number of histological specimens and heterogenous bone regeneration even within the individual groups, no measurable influence of adjuvant therapy with NO-coxib on the investigated markers could be observed.

To distinguish mature (type I) and immature (type II/III) collagen, in representative samples, Herovici’s Collagen stain for collagen differentiation (Figure S7A) as well as staining with antibodies specific for collagen I (Figure S7B) or collagen II (Figure S7C) was performed. Whereas collagen I could be detected in the entire bone defect, alongside with the presence of osteoblasts (Figure S7B,D), collagen II was restricted to areas filled with mature bone, as identified by Movat’s pentachrome stain (Figure S7C,E, arrows). Since collagen type II is specific for cartilage tissue, this can be interpreted as a marker for endochondrale ossification in these areas.

## 3. Discussion

Bone healing is a complex, multi-phase process that involves a series of cellular events aimed at restoring bone integrity following bone injury. It can be broken down into four main phases: inflammatory phase, soft callus formation, hard callus formation, and remodeling [39]. The inflammatory phase is initiated by tissue damage and blood vessel rupture that result in the formation of a hematoma at the injury site. This hematoma triggers an inflammatory response, with immune cells, e.g., macrophages, clearing debris and pathogens. Macrophages secrete cytokines and growth factors that initiate the healing process. During chondrogenesis, fibroblasts and chondrocytes migrate to the fracture site, forming a cartilage matrix (“soft callus”) that bridges the bone defect. Mesenchymal stroma cells (MSCs) differentiate into chondrocytes and osteoblasts, promoting the formation of periosteal bone. MSC-derived osteoblasts replace the cartilage with woven bone through a process known as endochondral ossification. The resulting “hard callus” provides temporary stability. Osteoclasts resorb the cartilage and mineralized bone to reshape and organize the newly formed tissue. The final remodeling phase involves the gradual replacement of woven bone with lamellar bone, restoring the bone’s original structure and strength. This last phase is driven by bone-forming osteoblasts and bone-resorbing osteoclasts, refining the bone’s architecture and adapting its strength to the mechanical load.

COX-2 represents a key factor in inflammatory processes accompanying bone healing by promoting the recruitment of cells required for proper remodeling, e.g., osteoclasts and osteoblasts [17,18,19]. While an appropriate course of inflammatory processes is essential for bone repair, excessive inflammation can interfere with healing. Therefore, reducing excessive inflammation by coxibs could potentially improve bone healing, particularly in situations of chronic inflammation. However, therapeutic modulation of COX-2 function is discussed controversially with regard to bone healing, especially during its early phase, because of the narrow therapeutic window [16,40]. Nevertheless, since COX-2 plays an essential role during bone healing, we hypothesize that targeting of COX-2 for the delivery of NO positively influences (biomaterial-assisted) bone healing. The importance of NO for bone function is well known. It is involved in neovascularization, delivery of inflammatory cells and cytokines to the fracture site [22,23,24], production of collagen precursors [20,23,25,26], as well as in appropriate osteoblast development [41]. In line with this, enhanced bone healing was achieved in a 5 mm rat femur defect model filled with demineralized bone matrix when the NO donor nitroso-bovine serum albumin (NO-BSA) was added directly to the implanted bone matrix [42]. Interestingly, the effect could be achieved by only a single dose of NO-BSA despite its short half-life of 5 h. It has been discussed that S-nitrosothiols, formed of NO and molecules containing sulfhydryl functional groups, e.g., serum albumin, retain the biological effectiveness of NO due to their substantially longer half-life compared to NO, resulting in a slow but continuous release of NO to the plasma [42]. In line with this, binding of NO-releasing moiety to lipophilic compounds like coxibs is hypothesized to enable prolonged release of NO.

Moreover, it has been shown in vitro that NO-releasing derivatives of aspirin (NCX-4016) and naproxen (HC-3012), by contrast to their parental compound aspirin and naproxen, respectively, or celecoxib, do not alter proliferation and differentiation of osteoblasts. Moreover, these NO-NSAIDs reduced the activity of matrix-modifying enzymes relevant for bone remodeling, namely plasminogen activator, metalloproteinases, and cathepsin B. Again S-nitrosylation of molecules like albumin was discussed as the underlying pharmacological mechanism [43].

Although autologous bone grafting, reflected by the positive control in this study, is still the clinical gold standard for the treatment of large bone defects, implantation of adaptive biomaterials is required in a situation when autologous bone is not sufficient. Thanks to its good biocompatibility, biodegradability, and mechanical elasticity, the FDA-approved synthetic linear polyester PCL provides a robust basis for scaffold development in bone tissue engineering [44,45]. A better interaction with the surrounding tissue, resulting in an optimized scaffold bioactivity, can be achieved by modification of the PCL-scaffolds’ surface, for example with natural ECM components [45]. In this regard, immobilization of collagen (Col) together with glycosaminoglycans (GAGs) like chondroitin sulfate (CS) or hyaluronan (HA), which are important multifunctional components of the extracellular matrix (ECM) in bone [4,5,6], offers beneficial effects on tissue integration and bone healing by binding of growth factors and cytokines as well as by recruiting MSCs and supporting their differentiation [5,7,8,46]. Via integrin-mediated binding to its arginylglycylaspartic acid (RGD) sequence, collagen (Col) promotes adhesion and differentiation of osteoblasts and osteoclasts but also their precursors, e.g., MSCs [8,47,48,49]. Moreover, Col serves as a substrate for collagenases and matrix metalloproteinases and, thereby, increases bone remodeling [8]. The precise action of GAGs varies according to their structural composition, mainly their degree of sulfation and polymer length, both of them also changing during bone remodeling processes [50,51]. In the present study, we included the most promising GAGs found in previous studies [5,14] resulting in an intense recruitment of cells, most likely MSCs, osteoblasts, and macrophages to the defect area, which could be confirmed by a significantly increased [^18^F]FDG uptake in Col/CS and Col/sHA3 group. Nevertheless, this does not result in an increased [^18^F]fluoride accumulation as an indicator for bone mineralization. This is in line with the controversial discussion on how much osteoblast activity is involved in [^18^F]fluoride uptake [36,52,53]. Nevertheless, both the increased presence of CD68-positive inflammatory cells and osteoblasts in the Col/CS and Col/sHA3 groups was confirmed by immunohistochemistry. Maybe due to this already highly cell-promotive effect we, by contrast to Baldik et al. [42], could not observe a measurable beneficial bone healing by NO delivery. This is supported by the fact that there is a trend towards an increased [^18^F]fluoride uptake in the groups without aECM-coated PCL-scaffolds, namely our negative and positive control, when rats were treated with NO-coxib. Following an injury, COX-2 expression increases within one day [54], which was the reason to start COX-2-targeted delivery of NO-coxib at day 2 post-surgery. However, COX-2 expression can also last longer, depending on the individual bone healing progress [54]. In a previous study, we could confirm COX-2 expression in the same 5 mm critical size femur defect model even after 8 weeks, especially in defects treated with aECM-coated PCL-scaffolds [6]. The fact that we could not demonstrate any significant effects of the NO-coxib on bone healing may support the hypothesis that NO compensates the negative effects often observed with the use of coxibs [16,55]. Thereby, such a dual-drug approach could change the therapeutic window of coxibs in a way that they could be used for fine-tuning of inflammatory processes in bone healing. Further studies would then have to include dose optimization and determination of the most favorable treatment period.

Non-invasive imaging techniques are particularly suitable to longitudinally investigate bone healing on an intraindividual level [56,57]. Especially in preclinical research, deep insights into (biomaterial-assisted) bone healing could be obtained by the use of single photon emission tomography (SPECT) or PET imaging [58,59,60,61,62,63], which paves the way also to their clinical application in this field [64,65]. In comparison to the morphological information obtained by CT, PET using, e.g., [^18^F]fluoride provides a functional information about osteoblast activity and bone mineralization and, thereby, can also be of prognostic value [36]. Findings of in vivo imaging techniques, reaching 3D to 4D level, can be supported by 2D histology and immunohistochemistry, which still is the gold standard technique for quantitative evaluation of biomaterial degradation and bone formation, although limited by its invasive and destructive character. By contrast to histology, µCT imaging most likely underestimates the amount of newly formed, still non-mineralized bone tissue. A promising technique to bridge the gap between µCT and histology in visualization of the bone regeneration process is micromagnetic resonance imaging (µMRI) if used in combination with µCT [66]. Although µMRI shows a richer tissue contrast than µCT, it is very labor- and evaluation-intensive, which may limit its broad application.

## 4. Materials and Methods

### 4.1. Synthesis of NO-Coxib

**Figure 6:**
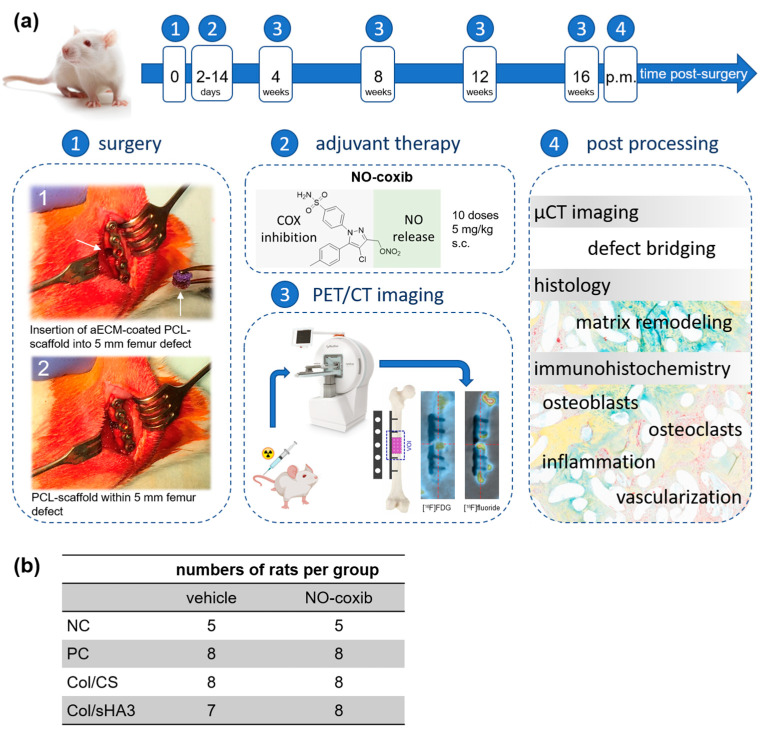
(**a**) Schematic overview of the study design. (1) The right femur was surgically exposed and a 5-hole plate was fixed with 4 screws. A 5 mm defect was created at the femur midshaft (upper arrow) using a wire saw. The defect was randomly filled with no material (negative control, NC), a mixture of collagen and autologous bone fragments (positive control, PC), or PCL-scaffolds (lower arrow) coated with a mixture of either collagen and chondroitin sulfate (Col/CS) or collagen and polysulfated hyaluronic acid (Col/sHA3). After adjuvant treatment with NO-coxib (2) bone defect healing was investigated by PET/CT imaging every 4 weeks for up to 16 weeks post-surgery (3). Tracer uptake was quantified in a volume of interest (VOI) comprising the bone defect and adjacent bone tissue between the two inner screws. At 16 weeks post-surgery, when rats passed all imaging experiments, animals were euthanized, femora were explanted and analyzed by ex vivo CT, histology, and immunohistochemistry to obtain insights into bone healing, remodeling, vascularization, and the presence of osteoblasts, osteoclasts, and inflammatory cells (4). (**b**) Number of rats within each group was comparable for adjuvant therapy with NO-coxib and vehicle injection (NC: 5; PC, Col/CS, Col/sHA3: 7–8).

[4-Chloro-1-(4-sulfamoylphenyl)-5-(p-tolyl)-1*H*-pyrazol-3-yl]methyl nitrate (NO-coxib, Figure 6a) was synthesized starting from methyl 1-(4-sulfamoylphenyl)-5-(4-tolyl)-1*H*-pyrazole-3-carboxylate following the previously established synthetic route [27,28]. In brief, methyl 1-(4-sulfamoylphenyl)-5-(4-tolyl)-1*H*-pyrazole-3-carboxylate (5.14 g, 13.84 mmol) was reacted under nitrogen atmosphere at room temperature with LiAlH_4_ (2.4 M in THF, 7.71 mL) in dry THF (250 mL) for 90 min, quenched with water (20 mL) and extracted from an acidified aqueous solution with EtOAc (4 × 300 mL). The combined organic phases were dried with Na_2_SO_4_, filtered, and evaporation under reduced pressure gave 4-[3-(hydroxymethyl)-5-(4-tolyl-1*H*-pyrazol-1-yl]benzenesulfonamide (4.72 g, 98.9%). Then, 4-[3-(hydroxymethyl)-5-(4-tolyl-1H-pyrazol-1-yl]benzenesulfonamide (4.72 g, 13.73 mmol) was reacted at 80 °C in dichloroethane (200 mL) with SOCl_2_ (addition of 6.54 g, 4.0 mL, 4 eq. at t = 0 h, t = 32 h, t = 48 h, and t = 72 h at room temperature) using UPLC-MS to follow reaction progress. After removal of SOCl_2_ and dichloroethane as reported, column chromatographic purification (silica gel, EtOAc/n-hexane 1/1) furnished 4-[4-chloro-3-(chloromethyl)-5-(4-tolyl)-1*H*-pyrazol-1-yl]benzenesulfonamide (2.20 g, 40%). Finally, 4-[4-chloro-3-(chloromethyl)-5-(4-toly)-1*H*-pyrazol-1-yl]benzenesulfonamide was reacted in batches (A: 902 mg, 2.28 mmol; B: 1200 mg, 3.03 mmol; C: 900 mg, 2.27 mmol; D: 800 mg, 2.02 mmol;) with AgNO_3_ (A: 1.16 g; B: 1.54 g, C: 1.15 g, D: 1.03 g, 3 eq.) in acetonitrile (A, C, D: 70 mL; B: 100 mL) by heating to reflux at 80 °C for 24 h under protection from light. Subsequent solvent evaporation, dissolution in EtOAc, filtration, evaporation, and purification by column chromatography (Biotage select SFär HC silica gel, acetone/n-hexane 29/71 → 47/53) provided crude NO-coxib (A: 954 mg; B: 1273 mg; C: 937 mg; D: 836 mg). Final column chromatographic purification (Biotage Select C18 60 g; gradient of acetonitrile/water from 16/84 → 100/0) of combined crude product (4.0 g) furnished analytically pure [4-chloro-1-(4-sulfamoylphenyl)-5-(p-tolyl)-1*H*-pyrazol-3-yl]methyl nitrate (NO-coxib) as white solid (2.58 g, 64%). Purity according to HPLC was monitored at 254 nm and found to be 98% for NO-coxib (Figure S8). Analytical data such as melting point (mp), retention factor (Rf) in thin-layer chromatography (TLC), data from nuclear magnetic resonance (NMR), and ultra-pressure liquid chromatography-mass spectrometry (UPLC-MS) obtained from intermediate compounds and NO-coxib as final product were in accordance with previous findings [27,28].

### 4.2. Rat Femur Defect Model and Adjuvant Therapy with NO-Coxib

All animal experiments were carried out according to the guidelines of the German Regulations for Animal Welfare. The protocols were approved by the local Ethical Committee for Animal Experiments (25-5131/449/39, date of approval: 9 September 2018). The femur defect model in male Wistar rats (Janvier Inc., Le Genest Saint Isle, France), aged 10–12 weeks and with an average weight of 300 g, was established as published elsewhere in detail [6]. In brief, a 5 mm femoral shaft defect was surgically created and bridged with a plate (Figure 6a) in 58 rats. Rats were randomly divided into four groups: (1) negative control (NC, defect was left empty), (2) positive control (PC, defect filled with a porcine gelatine consisting of collagen and sodium lauryl sulfate (spongostan^®^, Ethicon/Johnson & Johnson Medical GmbH, Norderstedt, Germany) and autologous bone fragments from the same femur) as well as group (3) and (4), where the femoral defect was filled with polycaprolactone-*co*-lactide (PCL)-scaffolds coated with either collagen and chondroitin sulfate (Col/CS) or collagen and polysulfated hyaluronan (Col/sHA3), respectively, as detailed elsewhere [6]. At 4 weeks post-surgery, one rat of the Col/sHA3 group had to be euthanized because of osteosynthesis failure found by PET/CT imaging.

For animal experiments, NO-coxib (Figure 6a) was dissolved in DMSO (50 mg/mL) and diluted 1:10 in a bio-compatible vehicle solution of 0.9% NaCl and PEG300 (*v*/*v* 4.5/5.5) to a concentration of 5 mg/mL for s.c. injection. Again, rats were randomly divided into two groups and treated with either NO-coxib solution (5 mg/kg, 1 mL/kg body weight) or the vehicle solution (50% PEG300/40% NaCl/10% DMSO, 1 mL/kg body weight). Rats were treated 10 out of 14 days from day 2 to day 14 post-surgery by s.c. injection of NO-coxib or vehicle solution next to the surgery area. The total number of animals within each group receiving either NO-coxib or vehicle solution are shown in Figure 6b.

### 4.3. In Vivo PET/CT Imaging and Data Processing

Bone healing was investigated intraindividually over 16 weeks by PET/CT imaging using the clinically established radiotracers 2-[^18^F]fluoro-2-deoxy-D-glucose ([^18^F]FDG) and [^18^F]fluoride (Figure 6a), both produced under GMP conditions at the Helmholtz-Zentrum Dresden-Rossendorf. Dual tracer PET/CT imaging and data processing essentially followed the protocol published elsewhere [6]. In brief, PET/CT imaging (nanoScan PET/CT, Mediso, Budapest, Hungary) was performed at 4, 8, 12, and 16 weeks post-surgery. PET/CT imaging is particularly suitable for such longitudinal studies as it allows for non-invasive imaging of the target of interest, which can be, depending on the type of used radiotracer, the expression of a certain receptor, activity of transporters or enzymes, or a metabolic process [29,30]. While [^18^F]FDG accumulates as a function of cellular glucose consumption and activity, and thereby reflects bone defect inflammation, [^18^F]fluoride uptake is a marker for osteoblast activity and bone mineralization.

Volumes of interest (VOI) for bone defect (VOI_defect_) as well as reference tissues blood (VOI_blood_) and muscle (VOI_muscle_) were determined using the software Rover (Version 3.0.61, ABX GmbH, Radeberg, Germany) with the fixed threshold method (percent of maximum radiotracer uptake) with manual adjustment, if necessary, at dedicated time frames with dedicated thresholds (VOI_defect_: 50–60 min p.i., 35%; VOI_muscle_: 30–60 min p.i., ≤45%; VOI_blood_: 0–80 s, 15%). Femur defect was confined by the two inner screws (VOI_defect_, Figure 6a). VOI_muscle_ was determined at the contralateral healthy leg and VOI_blood_ was placed over middle and lower third of vena cava. For all VOIs, the mean standardized uptake value (SUV_mean_ = mean activity concentration × body weight/injected dose) was computed and analyzed for a tracer kinetic-dependent time frame ([^18^F]FDG: 50–60 min p.i., [^18^F]fluoride: 50–60 min p.i.). In addition, SUV in bone defect was normalized to either tracer uptake in reference tissue muscle (target-to-background ratio; TBR) or tracer input function in blood (target-to-blood ratio; SUR [67]). Moreover, Patlak analysis [68] was performed to determine the metabolic uptake rate K_m_.

### 4.4. High-Resolution CT and µCT Imaging

After 16 weeks, when rats passed all imaging experiments, animals were sacrificed under desflurane anesthesia using CO_2_. Femora were resected and soft tissue as well as plate and screws were carefully removed. Femora were fixed by incubation in 4% buffered formaldehyde at 4 °C for at least 3 days. During this time, high-resolution CT imaging (35 kVp, 980 µmA, voxel size 39 µm) using nanoScan PET/CT system (Mediso, Budapest, Hungary) was performed to investigate femur defect bridging. In addition, µCT imaging (70 kVp, 114 µmA, voxel size 10.5 µm, 200 ms integration time) was carried out using vivaCT40 (Scanco Medical AG, Brüttisellen, Switzerland). While high-resolution CT data were analyzed by a scoring system, µCT data were used for determination of bone volume ratio (bone volume (BV)/total volume (TV)) at the femoral mid-diaphysis according to international guidelines [69].

### 4.5. Histology and Immunohistochemistry

For histological investigation, formalin-fixed femora were decalcified for 21 days with EDTA-based Osteosoft^®^ (Merck, Darmstadt, Germany). Afterwards, specimens were semi-automatically washed, dehydrated, and embedded in paraffin using the Thermo Scientific STP 420ES Tissue Processor (Microm International, Dreieich, Germany). Sequential sections (2 µm) were made in parallel to the longitudinal axis of the femur. Samples were stained with hematoxylin and eosin (HE; VWR, Darmstadt, Germany), Movat’s Pentachrome staining kit (Movat Pentachrom nach Verhöff, Morphisto, Offenbach am Main Germany), and Masson–Goldner staining kit (Gold; #1.00485, Merck, Darmstadt, Germany).

For immunohistochemical staining, bone specimens were incubated with antibodies against Tissue-Nonspecific Alkaline Phosphatase (TNAP; E-AB-93077, 1:200, Biozol, Hamburg, Germany), Tartrate-Resistant Acid Phosphatase (TRAP; GTX30018, 1:150, GeneTex, Irvine, CA, USA), α smooth muscle actin (α-SMA; M0851, 1:750, Agilent Dako, Santa Clara, CA, USA), CD68 (clone ED-1, MCA341R, 1:100, Bio-Rad, Feldkirchen, Germany), or CD163 (LS-C393444, 1:35, Lifespan Biosciences, Newark, CA, USA). Primary antibodies were detected with BrightVision Goat Anti-Mouse AP kit (for α-SMA and CD68; Medac, Wedel, Germany) or BrightVision Goat Anti-Rabbit AP kit (for TNAP, TRAP, and CD163; Medac, Wedel, Germany). Finally, immune reaction was visualized by Histofine DAB-2V (Medac, Wedel, Germany; for TNAP) or Permanent AP-Red Kit (Zytomed, Bargteheide, Germany).

### 4.6. Statistical Analysis

First, all data were subjected to a Dixon outlier test. Afterwards, statistical analysis was performed using software GraphPad Prism 9 (version 10.4.1, GraphPad Software, Boston, MA, USA). The effects of both the type of bone defect filling (NC vs. PC, Col/CS, or Col/sHA3) and the adjuvant treatment (NO-coxib vs. vehicle) at individual time points were estimated by two-way ANOVA with Geisser–Greenhouse correction and Bonferroni or Holm–Šídák post hoc test. Total tracer uptake over the entire observation period (AUC) was analyzed for significant differences with an unpaired T test. Statistical significance was set at *p* < 0.05.

## 5. Conclusions

With the in vivo and ex vivo approaches presented here, we aimed to investigate the essential hallmarks of bone defect healing in the presence of different biomaterials in combination with treatment by a novel NO-coxib. While in vivo PET/CT imaging allows for longitudinal investigation of functional markers like glucose consumption and bone mineralization, ex vivo analysis of bone specimens by CT imaging, histology, and immunohistochemistry is limited to a single time point per animal and, therefore, represents only one aspect of bone healing with regard to temporal and spatial resolution. However, these methods can give information up to a cellular level and, therefore, support in vivo investigations. By combining all of them, we could show that the delivery of NO to the defect area by targeting COX-2, a key molecular player during the inflammatory phase of bone healing, does not significantly influence bone healing in any of the experimental groups, reflecting different clinical situations. This supports the hypothesis that NO compensates for the negative effects of the coxib moiety and, thereby, opens the therapeutic window of coxibs for treatment of post-traumatic or post-operative pain and inflammation. As an example for biomaterial-assisted bone healing, PCL-scaffolds coated with either Col/CS or Col/sHA3 significantly increased the recruitment of cells, most likely osteoblasts and inflammatory cells, which could be demonstrated by both significantly higher [^18^F]FDG uptake in vivo and immunohistochemistry ex vivo. Non-invasive imaging techniques like PET are particularly suitable for such a longitudinal investigation of bone healing on an intraindividual level and, thereby, would be of added value also in clinical practice.

## Data Availability

The data presented in this study are contained within the article.

## Supplementary Materials

The supporting information can be downloaded at: https://www.mdpi.com/article/10.3390/ijms26062582/s1.

## References

[1] Bauer T.W., Muschler G.F. (2000). Bone graft materials. An overview of the basic science. Clin. Orthop. Relat. Res..

[2] Roddy E., DeBaun M.R., Daoud-Gray A., Yang Y.P., Gardner M.J. (2018). Treatment of critical-sized bone defects: Clinical and tissue engineering perspectives. Eur. J. Orthop. Surg. Traumatol..

[3] Albrektsson T., Johansson C. (2001). Osteoinduction, osteoconduction and osseointegration. Eur. Spine J..

[4] Buttner M., Moller S., Keller M., Huster D., Schiller J., Schnabelrauch M., Dieter P., Hempel U. (2013). Over-sulfated chondroitin sulfate derivatives induce osteogenic differentiation of hMSC independent of BMP-2 and TGF-beta1 signalling. J. Cell. Physiol..

[5] Förster Y., Bernhardt R., Hintze V., Moller S., Schnabelrauch M., Scharnweber D., Rammelt S. (2017). Collagen/glycosaminoglycan coatings enhance new bone formation in a critical size bone defect—A pilot study in rats. Mater. Sci. Eng. C Mater. Biol. Appl..

[6] Neuber C., Schulze S., Förster Y., Hofheinz F., Wodke J., Moller S., Schnabelrauch M., Hintze V., Scharnweber D., Rammelt S. (2019). Biomaterials in repairing rat femoral defects: In vivo insights from small animal positron emission tomography/computed tomography (PET/CT) studies. Clin. Hemorheol. Microcirc..

[7] Rammelt S., Illert T., Bierbaum S., Scharnweber D., Zwipp H., Schneiders W. (2006). Coating of titanium implants with collagen, RGD peptide and chondroitin sulfate. Biomaterials.

[8] Förster Y., Rentsch C., Schneiders W., Bernhardt R., Simon J.C., Worch H., Rammelt S. (2012). Surface modification of implants in long bone. Biomatter.

[9] Bierbaum S., Douglas T., Hanke T., Scharnweber D., Tippelt S., Monsees T.K., Funk R.H., Worch H. (2006). Collageneous matrix coatings on titanium implants modified with decorin and chondroitin sulfate: Characterization and influence on osteoblastic cells. J. Biomed. Mater. Res. A.

[10] Rentsch B., Hofmann A., Breier A., Rentsch C., Scharnweber D. (2009). Embroidered and surface modified polycaprolactone-co-lactide scaffolds as bone substitute: In vitro characterization. Ann. Biomed. Eng..

[11] Hempel U., Matthaus C., Preissler C., Moller S., Hintze V., Dieter P. (2014). Artificial matrices with high-sulfated glycosaminoglycans and collagen are anti-inflammatory and pro-osteogenic for human mesenchymal stromal cells. J. Cell. Biochem..

[12] Hempel U., Preissler C., Vogel S., Moller S., Hintze V., Becher J., Schnabelrauch M., Rauner M., Hofbauer L.C., Dieter P. (2014). Artificial extracellular matrices with oversulfated glycosaminoglycan derivatives promote the differentiation of osteoblast-precursor cells and premature osteoblasts. BioMed Res. Int..

[13] Stadlinger B., Bierbaum S., Grimmer S., Schulz M.C., Kuhlisch E., Scharnweber D., Eckelt U., Mai R. (2009). Increased bone formation around coated implants. J. Clin. Periodontol..

[14] Rentsch C., Rentsch B., Breier A., Spekl K., Jung R., Manthey S., Scharnweber D., Zwipp H., Biewener A. (2010). Long-bone critical-size defects treated with tissue-engineered polycaprolactone-co-lactide scaffolds: A pilot study on rats. J. Biomed. Mater. Res. A.

[15] Schulze S., Neuber C., Moller S., Pietzsch J., Schaser K.D., Rammelt S. (2023). Microdialysis Reveals Anti-Inflammatory Effects of Sulfated Glycosaminoglycanes in the Early Phase of Bone Healing. Int. J. Mol. Sci..

[16] Rothe R., Schulze S., Neuber C., Hauser S., Rammelt S., Pietzsch J. (2019). Adjuvant drug-assisted bone healing: Part I—Modulation of inflammation. Clin. Hemorheol. Microcirc..

[17] Zhang X.P., Schwarz E.M., Young D.A., Puzas J.E., Rosier R.N., O’Keefe R.J. (2002). Cyclooxygenase-2 regulates mesenchymal cell differentiation into the osteoblast lineage and is critically involved in bone repair. J. Clin. Investig..

[18] Lin H.N., O’Connor J.P. (2014). Immunohistochemical localization of key arachidonic acid metabolism enzymes during fracture healing in mice. PLoS ONE.

[19] Lu L.Y., Loi F., Nathan K., Lin T.H., Pajarinen J., Gibon E., Nabeshima A., Cordova L., Jamsen E., Yao Z. (2017). Pro-inflammatory M1 macrophages promote Osteogenesis by mesenchymal stem cells via the COX-2-prostaglandin E2 pathway. J. Orthop. Res..

[20] Anastasio A.T., Paniagua A., Diamond C., Ferlauto H.R., Fernandez-Moure J.S. (2020). Nanomaterial Nitric Oxide Delivery in Traumatic Orthopedic Regenerative Medicine. Front. Bioeng. Biotechnol..

[21] Rothe R., Schulze S., Neuber C., Hauser S., Rammelt S., Pietzsch J. (2019). Adjuvant drug-assisted bone healing: Part II—Modulation of angiogenesis. Clin. Hemorheol. Microcirc..

[22] Tomlinson R.E., Shoghi K.I., Silva M.J. (2014). Nitric oxide-mediated vasodilation increases blood flow during the early stages of stress fracture healing. J. Appl. Physiol..

[23] Ding Z.C., Lin Y.K., Gan Y.K., Tang T.T. (2018). Molecular pathogenesis of fracture nonunion. J. Orthop. Transl..

[24] Corbett S.A., McCarthy I.D., Batten J., Hukkanen M., Polak J.M., Hughes S.P. (1999). Nitric oxide mediated vasoreactivity during fracture repair. Clin. Orthop. Relat. Res..

[25] Meesters D.M., Neubert S., Wijnands K.A.P., Heyer F.L., Zeiter S., Ito K., Brink P.R.G., Poeze M. (2016). Deficiency of inducible and endothelial nitric oxide synthase results in diminished bone formation and delayed union and nonunion development. Bone.

[26] Corbett S.A., Hukkanen M., Batten J., McCarthy I.D., Polak J.M., Hughes S.P. (1999). Nitric oxide in fracture repair. Differential localisation, expression and activity of nitric oxide synthases. J. Bone Jt. Surg. Br..

[27] Brandt F., Ullrich M., Seifert V., Haase-Kohn C., Richter S., Kniess T., Pietzsch J., Laube M. (2022). Exploring Nitric Oxide (NO)-Releasing Celecoxib Derivatives as Modulators of Radioresponse in Pheochromocytoma Cells. Molecules.

[28] Bechmann N., Kniess T., Kockerling M., Pigorsch A., Steinbach J., Pietzsch J. (2015). Novel (pyrazolyl)benzenesulfonamides with a nitric oxide-releasing moiety as selective cyclooxygenase-2 inhibitors. Bioorg. Med. Chem. Lett..

[29] Phelps M.E. (1991). PET: A biological imaging technique. Neurochem. Res..

[30] van den Hoff J. (2005). Principles of quantitative positron emission tomography. Amino Acids.

[31] Rogasch J.M.M., Hofheinz F., van Heek L., Voltin C.A., Boellaard R., Kobe C. (2022). Influences on PET Quantification and Interpretation. Diagnostics.

[32] Warburg O., Posener K., Negelein E. (1924). On the metabolism of carcinoma cells. Biochem. Z..

[33] Sambuceti G., Cossu V., Bauckneht M., Morbelli S., Orengo A., Carta S., Ravera S., Bruno S., Marini C. (2021). ^18^F-fluoro-2-deoxy-d-glucose (FDG) uptake. What are we looking at?. Eur. J. Nucl. Med. Mol. Imaging.

[34] Wu C., Li F., Niu G., Chen X. (2013). PET imaging of inflammation biomarkers. Theranostics.

[35] Hawkins R.A., Choi Y., Huang S.C., Hoh C.K., Dahlbom M., Schiepers C., Satyamurthy N., Barrio J.R., Phelps M.E. (1992). Evaluation of the Skeletal Kinetics of Fluorine-18-Fluoride Ion with PET. J. Nucl. Med..

[36] Mathavan N., Koopman J., Raina D.B., Turkiewicz A., Tagil M., Isaksson H. (2019). ^18^F-fluoride as a prognostic indicator of bone regeneration. Acta Biomater..

[37] Rentsch C., Schneiders W., Manthey S., Rentsch B., Rammelt S. (2014). Comprehensive histological evaluation of bone implants. Biomatter.

[38] Vimalraj S. (2020). Alkaline phosphatase: Structure, expression and its function in bone mineralization. Gene.

[39] Einhorn T.A., Gerstenfeld L.C. (2015). Fracture healing: Mechanisms and interventions. Nat. Rev. Rheumatol..

[40] Seidenberg A.B., An Y.H. (2004). Is there an inhibitory effect of COX-2 inhibitors on bone healing?. Pharmacol. Res..

[41] Saura M., Tarin C., Zaragoza C. (2010). Recent insights into the implication of nitric oxide in osteoblast differentiation and proliferation during bone development. Sci. World J..

[42] Baldik Y., Talu U., Altinel L., Bilge H., Demiryont M., Aykac-Toker G. (2002). Bone healing regulated by nitric oxide: An experimental study in rats. Clin. Orthop. Relat. Res..

[43] Aisa M.C., Datti A., Orlacchio A., Di Renzo G.C. (2018). COX inhibitors and bone: A safer impact on osteoblasts by NO-releasing NSAIDs. Life Sci..

[44] Todd E.A., Mirsky N.A., Silva B.L.G., Shinde A.R., Arakelians A.R.L., Nayak V.V., Marcantonio R.A.C., Gupta N., Witek L., Coelho P.G. (2024). Functional Scaffolds for Bone Tissue Regeneration: A Comprehensive Review of Materials, Methods, and Future Directions. J. Funct. Biomater..

[45] Abdal-hay A., Sheikh F.A., Gómez-Cerezo N., Alneairi A., Luqman M., Pant H.R., Ivanovski S. (2022). A review of protein adsorption and bioactivity characteristics of poly ε-caprolactone scaffolds in regenerative medicine. Eur. Polym. J..

[46] Ruoslahti E., Yamaguchi Y. (1991). Proteoglycans as modulators of growth factor activities. Cell.

[47] Ruoslahti E. (1989). Proteoglycans in cell regulation. J. Biol. Chem..

[48] Brighton C.T., Albelda S.M. (1992). Identification of integrin cell-substratum adhesion receptors on cultured rat bone cells. J. Orthop. Res..

[49] Salasznyk R.M., Williams W.A., Boskey A., Batorsky A., Plopper G.E. (2004). Adhesion to Vitronectin and Collagen I Promotes Osteogenic Differentiation of Human Mesenchymal Stem Cells. BioMed Res. Int..

[50] Salbach J., Kliemt S., Rauner M., Rachner T.D., Goettsch C., Kalkhof S., von Bergen M., Moller S., Schnabelrauch M., Hintze V. (2012). The effect of the degree of sulfation of glycosaminoglycans on osteoclast function and signaling pathways. Biomaterials.

[51] Salbach J., Rachner T.D., Rauner M., Hempel U., Anderegg U., Franz S., Simon J.C., Hofbauer L.C. (2012). Regenerative potential of glycosaminoglycans for skin and bone. J. Mol. Med..

[52] Toegel S., Hoffmann O., Wadsak W., Ettlinger D., Mien L.K., Wiesner K., Nguemo J., Viernstein H., Kletter K., Dudczak R. (2006). Uptake of bone-seekers is solely associated with mineralisation! A study with Tc-99m-MDP, Sm-153-EDTMP and F-18-fluoride on osteoblasts. Eur. J. Nucl. Med. Mol. Imaging.

[53] Sorensen J., Ullmark G. (2009). PET scanning for evaluation of bone metabolism. Acta Orthop..

[54] Volaric D., Zauhar G., Chen J., Jerbic Radetic A.T., Omrcen H., Raic A., Pirovic R., Cvijanovic Peloza O. (2024). The Effect of Low-Intensity Pulsed Ultrasound on Bone Regeneration and the Expression of Osterix and Cyclooxygenase-2 during Critical-Size Bone Defect Repair. Int. J. Mol. Sci..

[55] Cottrell J., O’Connor J.P. (2010). Effect of Non-Steroidal Anti-Inflammatory Drugs on Bone Healing. Pharmaceuticals.

[56] Tremoleda J.L., Khalil M., Gompels L.L., Wylezinska-Arridge M., Vincent T., Gsell W. (2011). Imaging technologies for preclinical models of bone and joint disorders. EJNMMI Res..

[57] Ventura M., Boerman O.C., de Korte C., Rijpkema M., Heerschap A., Oosterwijk E., Jansen J.A., Walboomers X.F. (2014). Preclinical imaging in bone tissue engineering. Tissue Eng. Part. B Rev..

[58] Ventura M., Franssen G.M., Oosterwijk E., Boerman O.C., Jansen J.A., Walboomers X.F. (2016). SPECT vs. PET monitoring of bone defect healing and biomaterial performance in vivo. J. Tissue Eng. Regen. Med..

[59] Cheng C., Alt V., Dimitrakopoulou-Strauss A., Pan L., Thormann U., Schnettler R., Weber K., Strauss L.G. (2013). Evaluation of new bone formation in normal and osteoporotic rats with a 3-mm femur defect: Functional assessment with dynamic PET-CT (dPET-CT) using 2-deoxy-2-[^18^F]fluoro-D-glucose (^18^F-FDG) and ^18^F-fluoride. Mol. Imaging Biol..

[60] Cheng C., Alt V., Pan L., Thormann U., Schnettler R., Strauss L.G., Schumacher M., Gelinsky M., Dimitrakopoulou-Strauss A. (2014). Preliminary evaluation of different biomaterials for defect healing in an experimental osteoporotic rat model with dynamic PET-CT (dPET-CT) using F-18-sodium fluoride (NaF). Injury.

[61] Cheng C., Alt V., Pan L., Thormann U., Schnettler R., Strauss L.G., Heinemann S., Schumacher M., Gelinsky M., Nies B. (2014). Application of F-18-sodium fluoride (NaF) dynamic PET-CT (dPET-CT) for defect healing: A comparison of biomaterials in an experimental osteoporotic rat model. Med. Sci. Monit..

[62] Schar M.O., Ma R., Demange M., Morgan M., Chen T., Ballon D.J., Dyke J.P., Deng X.H., Rodeo S.A. (2022). Use of small animal PET-CT imaging for in vivo assessment of tendon-to-bone healing: A pilot study. J. Orthop. Surg..

[63] Schulze S., Rothe R., Neuber C., Hauser S., Ullrich M., Pietzsch J., Rammelt S. (2021). Men who stare at bone: Multimodal monitoring of bone healing. Biol. Chem..

[64] Petri M., Namazian A., Wilke F., Ettinger M., Stubig T., Brand S., Bengel F., Krettek C., Berding G., Jagodzinski M. (2013). Repair of segmental long-bone defects by stem cell concentrate augmented scaffolds: A clinical and positron emission tomography--computed tomography analysis. Int. Orthop..

[65] Lundblad H., Karlsson-Thur C., Maguire G.Q., Jonsson C., Noz M.E., Zeleznik M.P., Weidenhielm L. (2017). Can Spatiotemporal Fluoride (^18^F^−^) Uptake be Used to Assess Bone Formation in the Tibia? A Longitudinal Study Using PET/CT. Clin. Orthop. Relat. Res..

[66] Sinibaldi R., Conti A., Sinjari B., Spadone S., Pecci R., Palombo M., Komlev V.S., Ortore M.G., Tromba G., Capuani S. (2018). Multimodal-3D imaging based on muMRI and muCT techniques bridges the gap with histology in visualization of the bone regeneration process. J. Tissue Eng. Regen. Med..

[67] Hofheinz F., Hoff J., Steffen I.G., Lougovski A., Ego K., Amthauer H., Apostolova I. (2016). Comparative evaluation of SUV, tumor-to-blood standard uptake ratio (SUR), and dual time point measurements for assessment of the metabolic uptake rate in FDG PET. EJNMMI Res..

[68] Patlak C.S., Blasberg R.G. (1985). Graphical Evaluation of Blood-to-Brain Transfer Constants from Multiple-Time Uptake Data-Generalizations. J. Cereb. Blood Flow Metab..

[69] Bouxsein M.L., Boyd S.K., Christiansen B.A., Guldberg R.E., Jepsen K.J., Muller R. (2010). Guidelines for assessment of bone microstructure in rodents using micro-computed tomography. J. Bone Miner. Res..

