# Supplementary material for: Exploring a Nitric Oxide-Releasing Celecoxib Derivative as a Potential Modulator of Bone Healing: Insights from Ex Vivo and In Vivo Imaging Experiments
Source: Int J Mol Sci. 2025 Mar 13;26(6):2582. doi: 10.3390/ijms26062582 (PMC11942287; doi:10.3390/ijms26062582)
Supplement: Supplementary file 1 [file ijms-26-02582-s001.zip › ijms-3475126-supplementary.pdf]

International Journal of
Molecular Sciences

MDPI

Supplementary Materials

Exploring a nitric oxide-releasing celecoxib derivative as a potential modulator of bone healing: insights from ex vivo and in vivo imaging experiments

Christin Neuber [1,*], Luisa Niedenzu [2], Sabine Schulze [2], Markus Laube [1], Frank Hofheinz [3], Stefan Rammelt [2] and Jens Pietzsch [1,4]

1 Department Radiopharmaceutical and Chemical Biology, Institute of Radiopharmaceutical Cancer Research, Helmholtz-Zentrum Dresden-Rossendorf, Bautzner Landstrasse 400, 01328 Dresden, Germany;
m.laube@hzdr.de (M.L.); j.pietzsch@hzdr.de (J.P.)
2 University Center for Orthopaedics, Trauma and Plastic Surgery, University Hospital Carl Gustav Carus at Technische Universität Dresden, Fetscherstrasse 74, 01307 Dresden, Germany; sabine.schulze@tu-dresden.de (S.S.); stefan.rammelt@ukdd.de (S.R.)
3 Department Positron Emission Tomography, Institute of Radiopharmaceutical Cancer Research, Helmholtz-Zentrum Dresden-Rossendorf, Bautzner Landstrasse 400, 01328 Dresden, Germany;
f.hofheinz@hzdr.de (F.H.)
4 Faculty of Chemistry and Food Chemistry, School of Science, Technische Universität Dresden, Bergstraße 66, 01069 Dresden, Germany

* Correspondence: c.neuber@hzdr.de

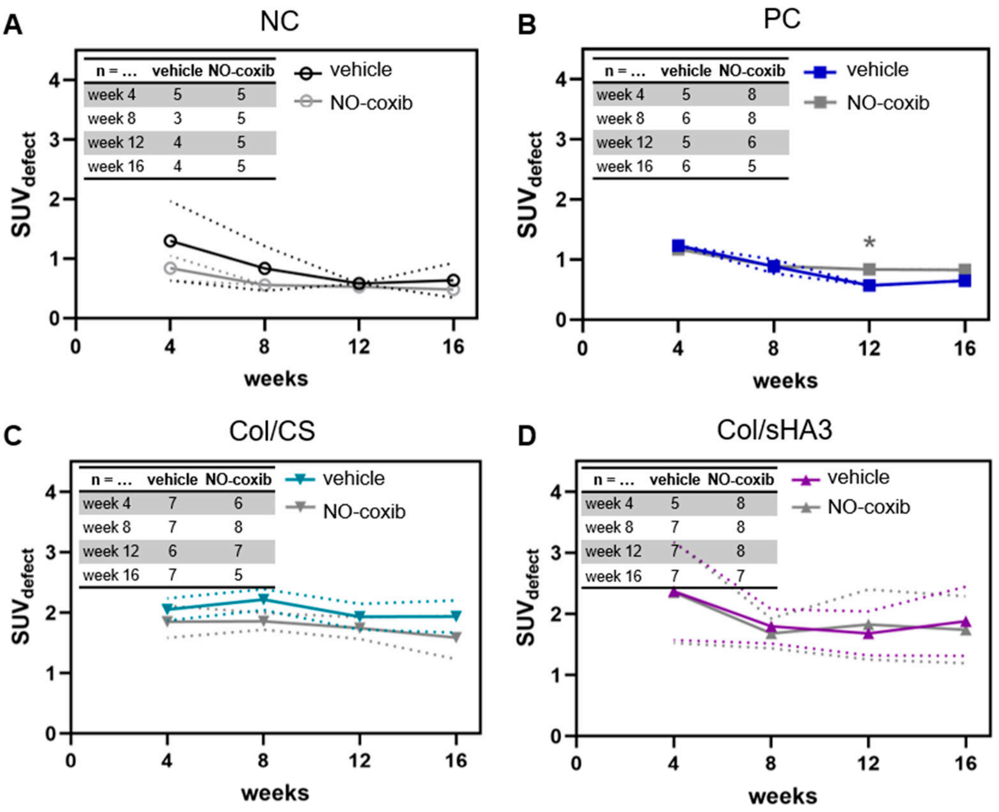

E

AUC_{SUV}	vehicle	NO-coxib	p =
NC	9.6 ± 1.8	7.0 ± 0.5	0.115
PC	9.6 ± 0.9	11.0 ± 0.8	0.362
Col/CS	24.6 ± 2.6	21.3 ± 2.7	0.373
Col/sHA3	22.5 ± 2.4	22.2 ± 2.7	0.954

$p \leq 0.01$

Figure S1. [18F]FDG accumulation in the femur defect model after insertion of the different materials in vehicle vs. NO-coxib treated rats. **(A-D)** SUV_{defect} was analyzed in rats receiving vehicle solution only (coloured line) or adjuvant administration of NO-coxib (grey line). All values are depicted as mean (symbol) ± SEM (dotted line). Significant differences to the vehicle group are indicated by * ($p \leq 0.05$). **(E)** Total [18F]FDG uptake over time was determined by analyzing AUC_{SUV}. FDG = 2-[18F]fluoro-2-deoxy-D-glucose, NC = negative control, PC = positive control (collagen with autologous bone), Col/CS = PCL-scaffold coated with collagen and chondroitin sulfate, Col/sHA3 = PCL-scaffold coated with collagen and polysulfated hyaluronic acid.

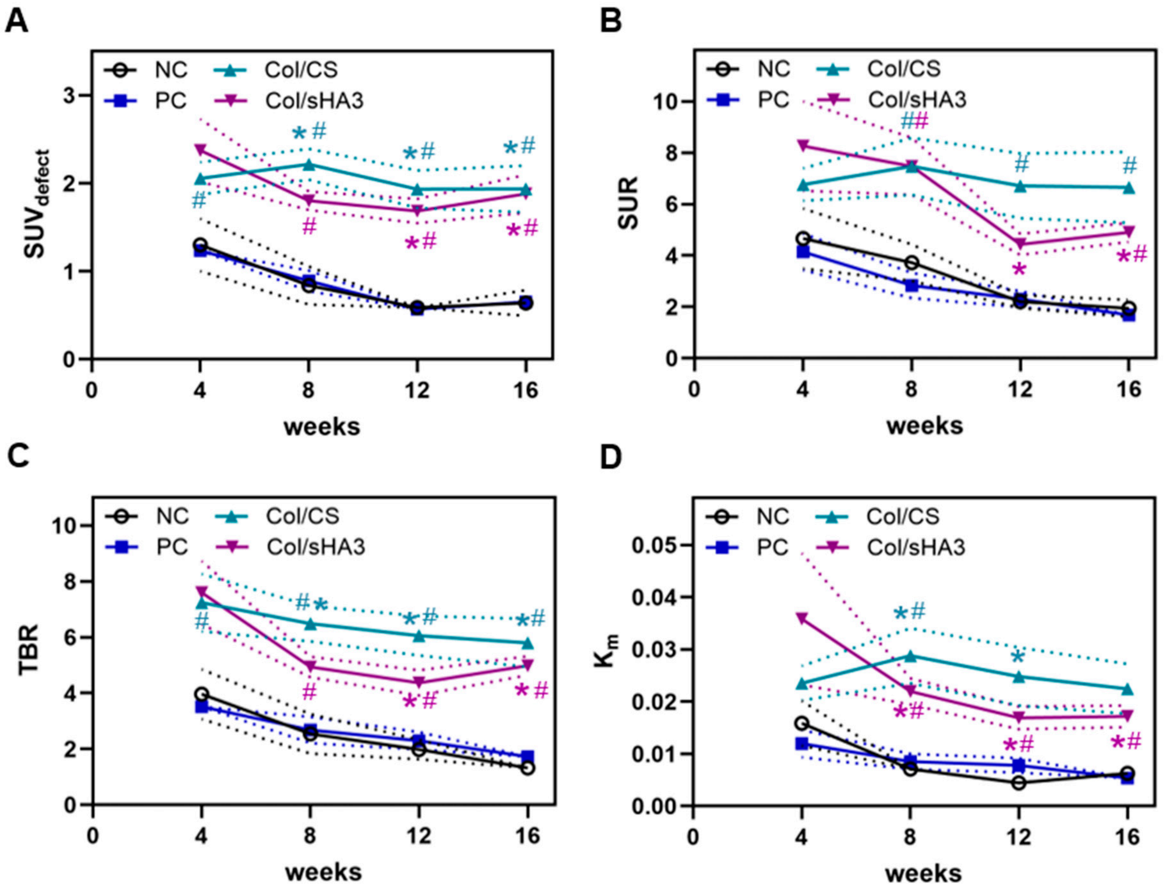

Figure S2. Comparison of **(A)** SUV, **(B)** SUR, **(C)** TBR, and **(D)** K_m to assess [18F]FDG accumulation in the femur defect model after insertion of the different materials. All values are depicted as mean (symbol) ± SEM (dotted line). Significant differences to the NC and PC are indicated by * and #, respectively (P≤0.05). SUV = standard uptake volume, SUR = standard uptake ratio, TBR = target-to-background ratio, K_m = metabolic uptake rate, FDG = 2-[18F]fluoro-2-deoxy-D-glucose, NC = negative control, PC = positive control (collagen with autologous bone), Col/CS = PCL-scaffold coated with collagen and chondroitin sulfate, Col/sHA3 = PCL scaffold coated with collagen and polysulfated hyaluronic acid.

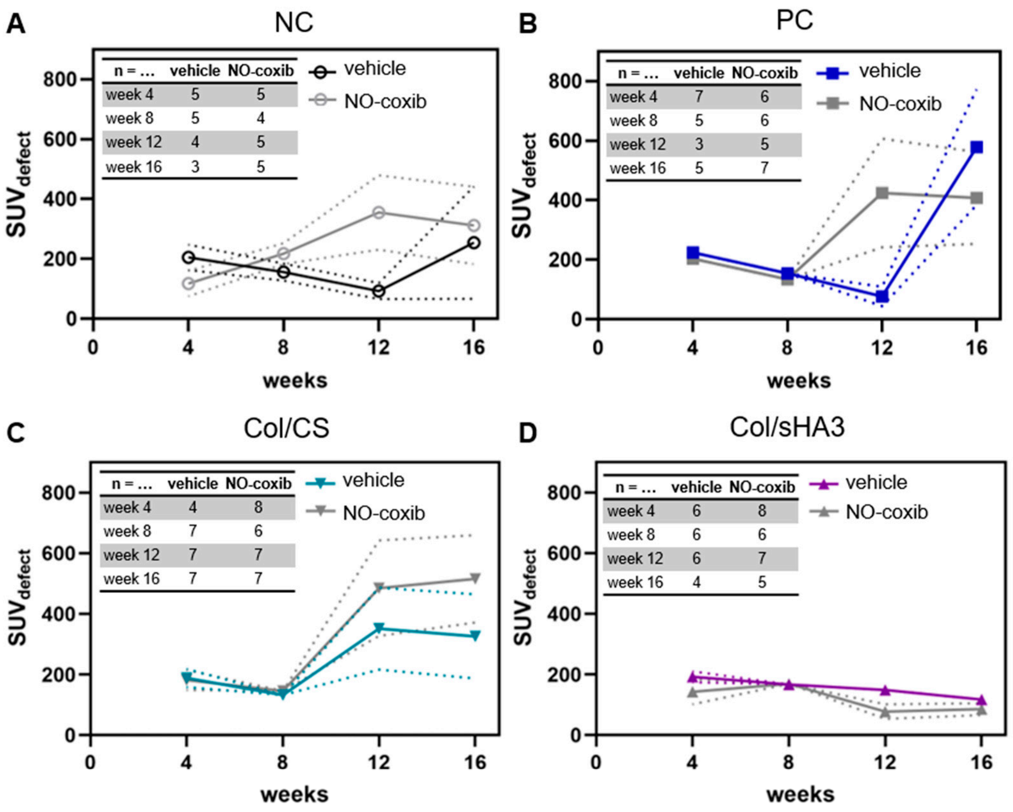

E

AUC_{SUV}	vehicle	NO-coxib	*p* =
NC	1917 ± 718	3155 ± 1016	0.057
PC	2533 ± 889	3456 ± 1421	0.171
Col/CS	2962 ± 1260	3918 ± 1419	0.194
Col/sHA3	1882 ± 176	1445 1± 323	0.007

Figure S3. [^{18}F]Fluoride accumulation in the femur defect model after insertion of the different materials in vehicle vs. NO-coxib treated rats. **(A-D)** SUV_{defect} was analyzed in rats receiving vehicle solution only (coloured line) or adjuvant administration of NO-coxib (grey line). All values are depicted as mean (symbol) ± SEM (dotted line). No significant differences to the vehicle group could be observed. **(E)** Total [^{18}F]fluoride uptake over time was determined by analyzing AUC_{SUV}. NC = negative control, PC = positive control (collagen with autologous bone), Col/CS = PCL scaffold coated with collagen and chondroitin sulfate, Col/sHA3sHA3 = PCL scaffold coated with collagen and polysulfated hyaluronic acid.

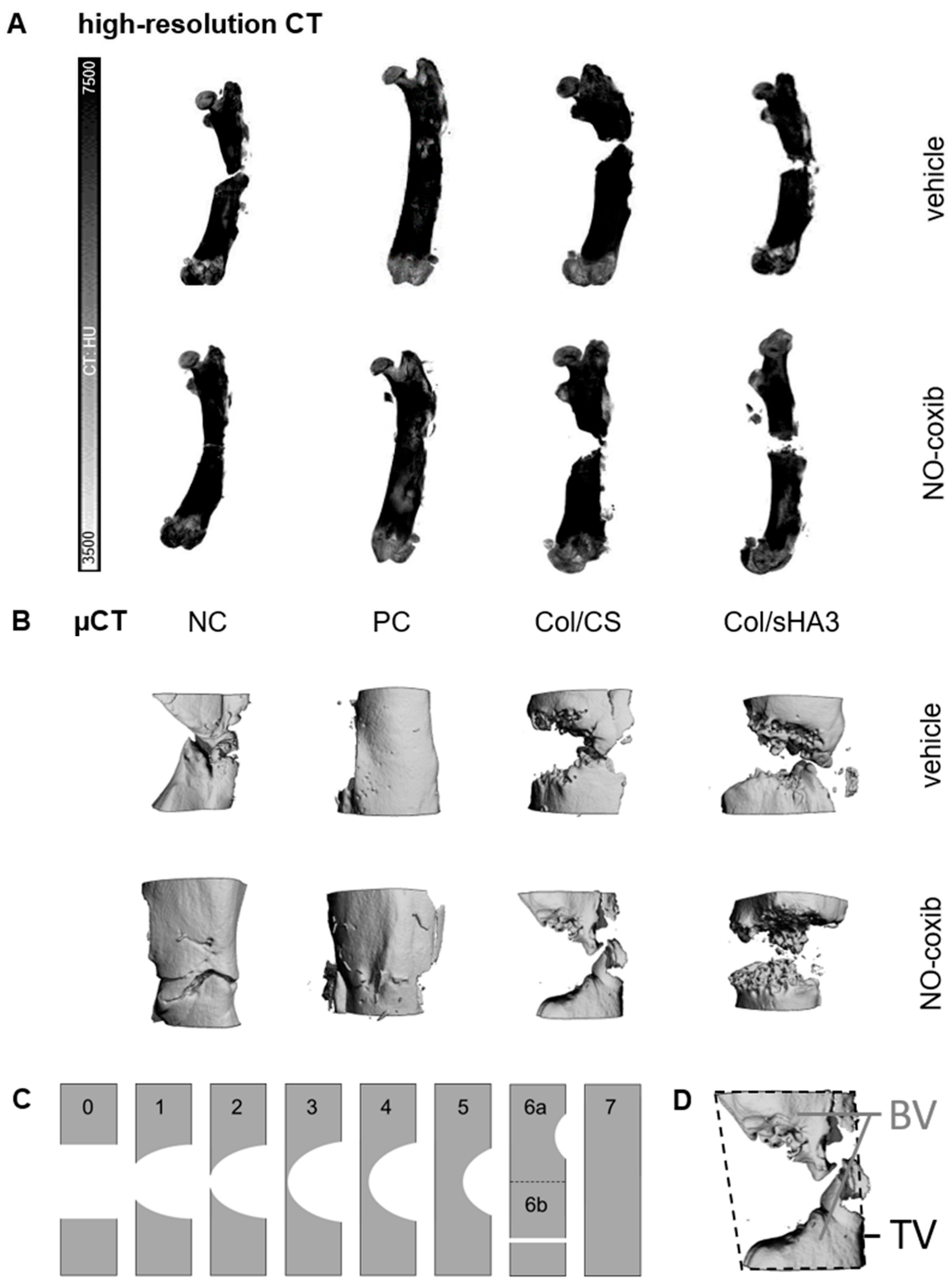

Figure S4. CT-based evaluation of defect bridging in explanted femora 16 weeks after induction of a 5 mm femur defect and insertion of the different materials. **(A)** Representative high-resolution CT images (voxel size 39 µm) of femurs directly after explantation. **(B)** Representative µCT images (voxel size 10.5 µm) of femurs after fixation. To investigate influence of different biomaterials and adjuvant administration of NO-coxib, extend of defect bridging was quantified using a defect closure scoring system **(C)** for high-resolution CT images or by software-based calculation of the ratio between bone and total volume (**D**; BV/TV) for µCT. CT = computed tomography, NO = nitric oxide, NC = negative control, PC = positive control (collagen with autologous bone), Col/CS = PCL-scaffold coated with collagen and chondroitin sulfate, Col/sHA3 = PCL-scaffold coated with collagen and polysulfated hyaluronic acid.

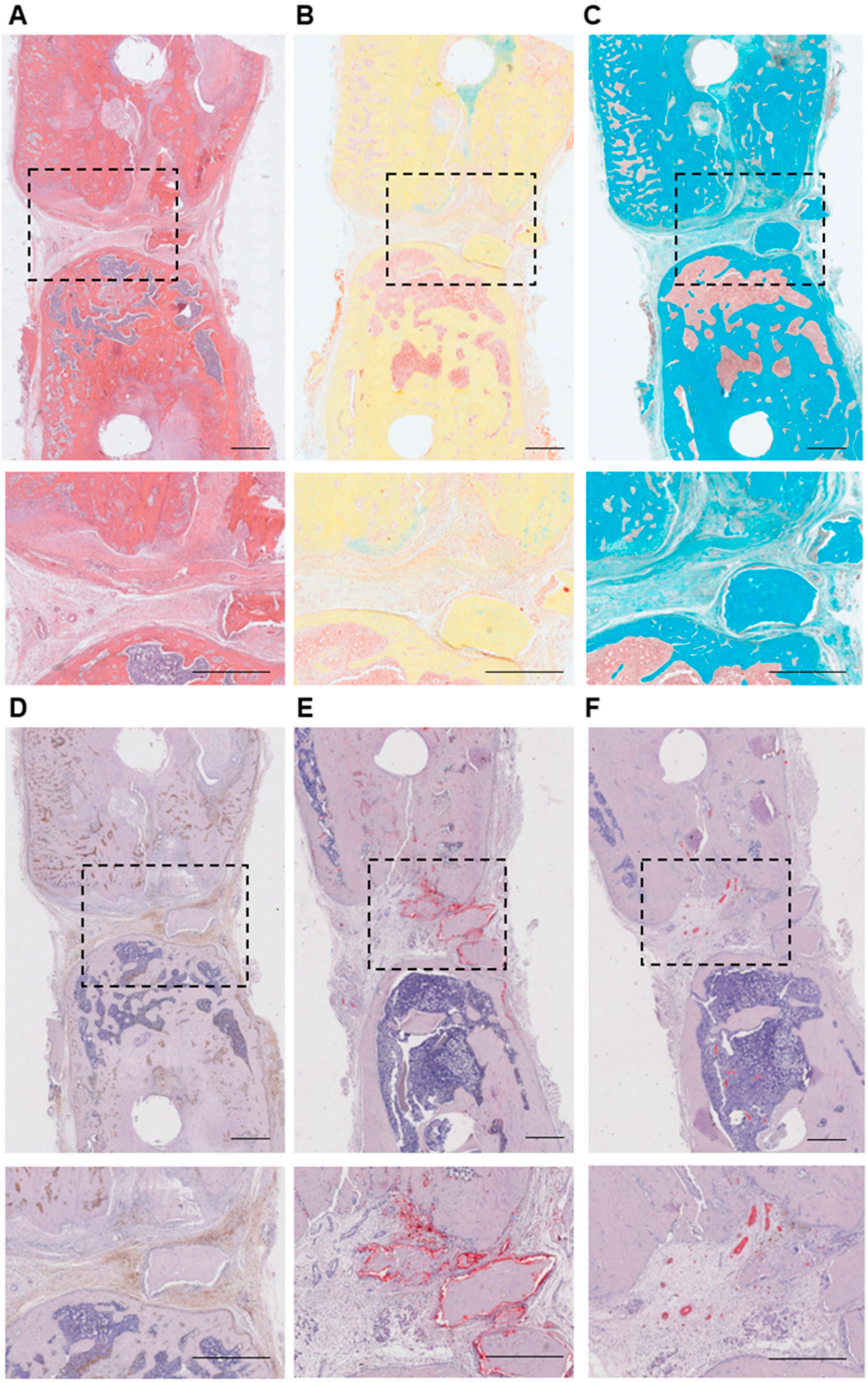

Figure S5. Representative images of **(A)** Hematoxylin and Eosin (HE), **(B)** Movat's pentachrome, and **(C)** Masson-Goldner trichrome stain of defect bridging in the femur defect model 8 weeks after insertion of collagen together with autologous bone (PC). Immunohistochemical staining of **(D)** osteoblasts (TNAP), **(E)** M1 macrophages (CD68), and **(F)** blood vascular endothelial cells (α-SMA). A representative section (dotted box) each is shown enlarged below. Bar = 1 cm

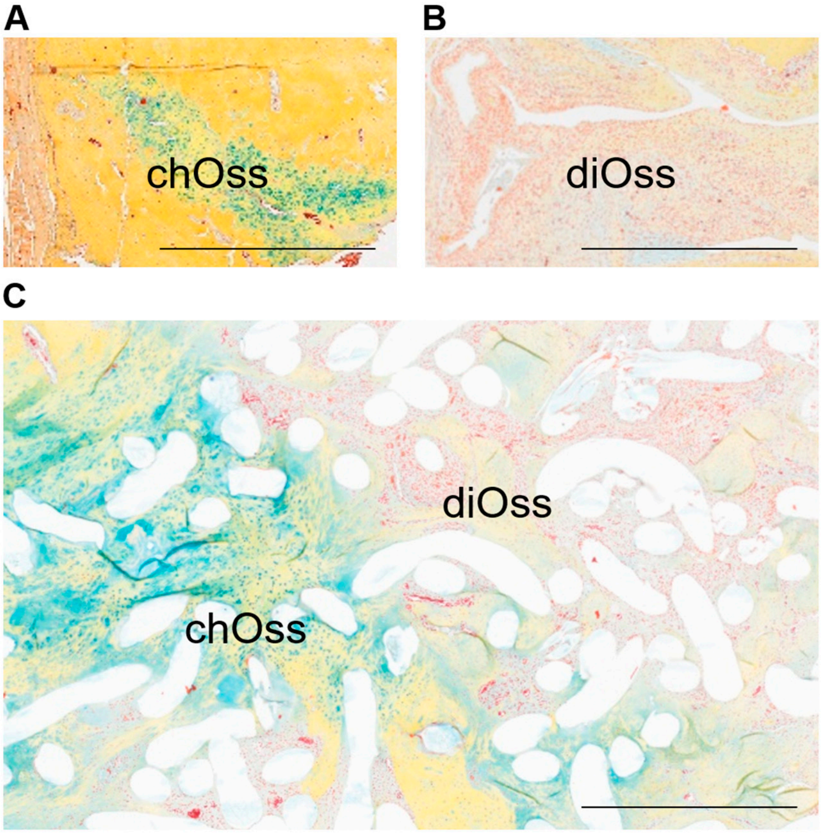

Figure S6. Differentiation of defect bridging into direct (diOss) and endochondral (chOss) ossification by Movat's pentachrome staining in femur defects filled with **(A)** no material (NC), **(B)** collagen together with autologous bone (PC), and **(C)** a PCL-scaffold coated with a mixture of collagen and chondroitin sulfate (Col/CS). Bar = 1 cm.

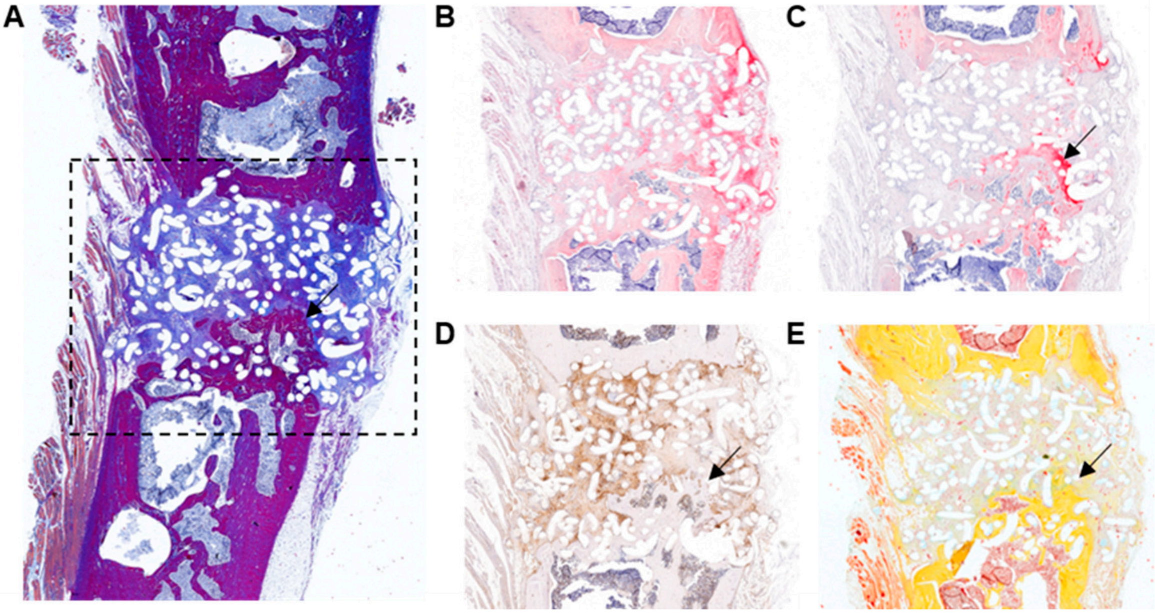

Figure S7. Representative images of Herovici's Collagen stain **(A)** and immunohistochemical staining of collagen type I **(B)**, collagen type II **(C)**, and TNAP **(D)** compared to Movat's pentachrome stain **(E)** in a representative section (dotted box) of femur defect 16 weeks after implantation of a PCL-scaffold coated with collagen and polysulfated hyaluronic acid (Col/sHA3). The arrows depict the area of newly formed bone within the defect that is subject to endochondral ossification.

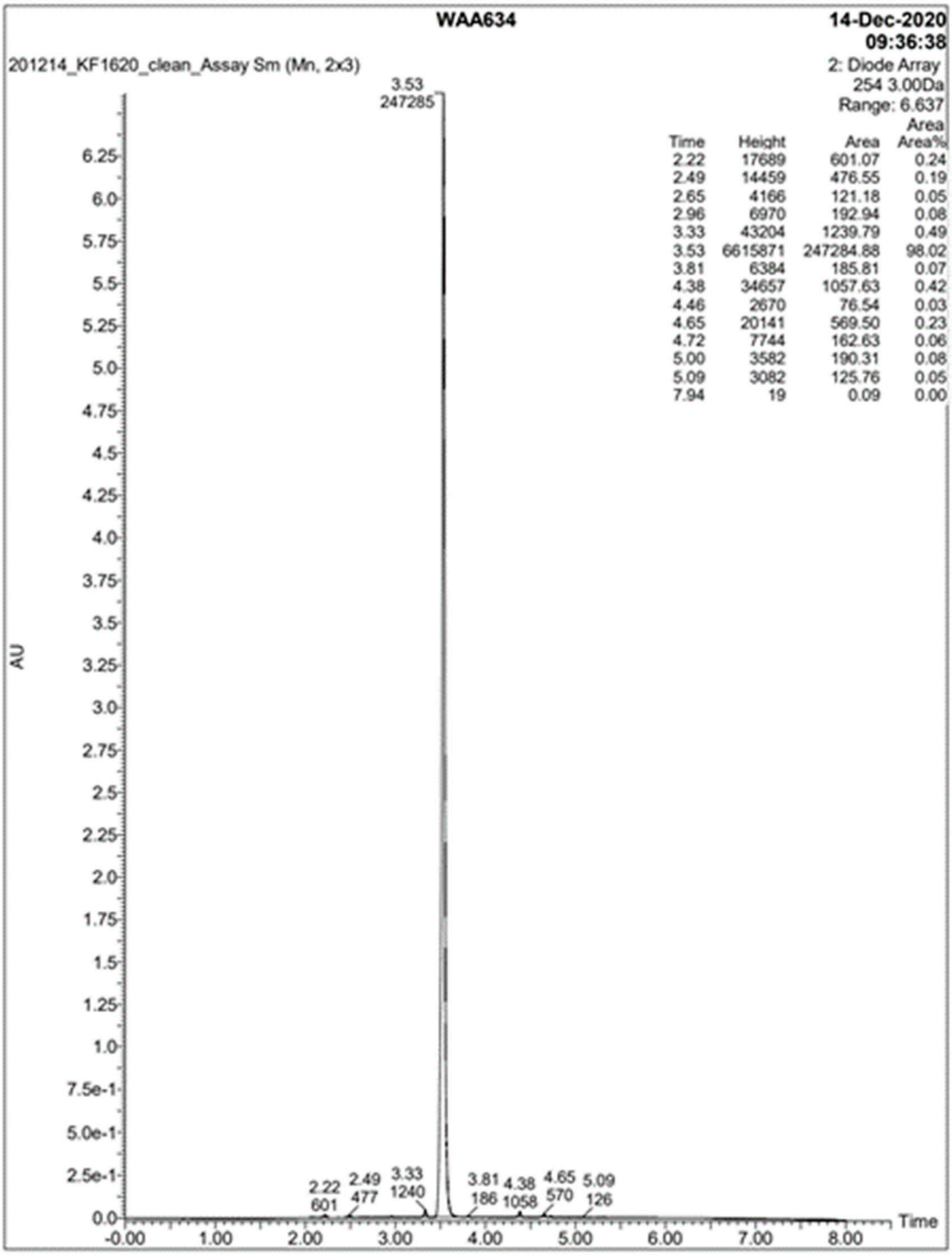

Figure S8. UPLC of NO-coxib monitored at 254 nm. Data were recorded with the following UPLC-MS-System: column Aquity UPLC® BEH C18 column (Waters Corporation, Milford, MA, USA, 100 × 2.1 mm, 1.7 µm, 130 Å), UPLC I-Class (Waters Corporation, Milford, MA, USA): binary gradient pump BSM, autosampler FTN, column manager CM, and diode array detector PDAeλ coupled to Waters Xevo TQ-S, flow rate 0.4 mL/min, eluent: (A): 0.1% acetic acid in MeCN/MeOH 1/1/ (B): 0.1% acetic acid in H_2O; gradient: t_{0min} 45/55–$t_{0.5min}$ 45/55–$t_{5.5min}$ 95/5–$t_{7.0min}$ 95/5–$t_{8.0min}$ 45/55–$t_{8.5min}$ 45/55).
